# Effect of perioperative goal-directed hemodynamic therapy on postoperative recovery following major abdominal surgery—a systematic review and meta-analysis of randomized controlled trials

**DOI:** 10.1186/s13054-017-1728-8

**Published:** 2017-06-12

**Authors:** Yanxia Sun, Fang Chai, Chuxiong Pan, Jamie Lee Romeiser, Tong J. Gan

**Affiliations:** 1 Department of Anesthesiology, Beijing Tong Ren Hospital, Capital Medical University, Beijing, 100730 China; 2 Department of Surgery, Stony Brook University, Stony Brook, NY USA; 3 Department of Anesthesiology, Stony Brook University, Stony Brook, NY USA

**Keywords:** Goal-directed hemodynamic therapy, Mortality, Morbidity, Gastrointestinal function, Abdominal surgery

## Abstract

**Background:**

Goal-directed hemodynamic therapy (GDHT) has been used in the clinical setting for years. However, the evidence for the beneficial effect of GDHT on postoperative recovery remains inconsistent. The aim of this systematic review and meta-analysis was to evaluate the effect of perioperative GDHT in comparison with conventional fluid therapy on postoperative recovery in adults undergoing major abdominal surgery.

**Methods:**

Randomized controlled trials (RCTs) in which researchers evaluated the effect of perioperative use of GDHT on postoperative recovery in comparison with conventional fluid therapy following abdominal surgery in adults (i.e., >16 years) were considered. The effect sizes with 95% CIs were calculated.

**Results:**

Forty-five eligible RCTs were included. Perioperative GDHT was associated with a significant reduction in short-term mortality (risk ratio [RR] 0.75, 95% CI 0.61–0.91, *p* = 0.004, *I* ^2^ = 0), long-term mortality (RR 0.80, 95% CI 0.64–0.99, *p* = 0.04, *I* ^2^ = 4%), and overall complication rates (RR 0.76, 95% CI 0.68–0.85, *p* < 0.0001, *I* ^2^ = 38%). GDHT also facilitated gastrointestinal function recovery, as demonstrated by shortening the time to first flatus by 0.4 days (95% CI −0.72 to −0.08, *p* = 0.01, *I* ^2^ = 74%) and the time to toleration of oral diet by 0.74 days (95% CI −1.44 to −0.03, *p* < 0.0001, *I* ^2^ = 92%).

**Conclusions:**

This systematic review of available evidence suggests that the use of perioperative GDHT may facilitate recovery in patients undergoing major abdominal surgery.

**Electronic supplementary material:**

The online version of this article (doi:10.1186/s13054-017-1728-8) contains supplementary material, which is available to authorized users.

## Background

Perioperative fluid management has been recognized as an important factor in postoperative recovery following major abdominal surgery [1, 2]. There is evidence that either too little or too much fluid administration during the perioperative period was associated with organ dysfunction, delayed gastrointestinal (GI) function, and increased complication rates after surgery [3]. However, optimal fluid management is difficult to achieve using standard parameters (e.g., heart rate [HR], blood pressure [BP], central venous pressure [CVP], or urine output) that poorly estimate preload and preload responsiveness [4].

Goal-directed hemodynamic therapy (GDHT) was proposed by introducing different hemodynamic variables into a dynamic perspective of individual fluid loading with or without vasoactive substances to reach a predefined goal of optimal preload and/or oxygen delivery [5]. An increasing numbers of studies of the effect of perioperative GDHT on postoperative recovery following major abdominal surgery are being done. However, the evidence for the beneficial effect of GDHT on postoperative recovery remains inconsistent. Several meta-analyses demonstrated that GDHT could decrease postoperative morbidity and mortality in patients undergoing major surgery [1, 6, 7], but others suggested that the treatment benefit may be more marginal than previously believed [8–10]. More recent studies [1, 11–14] have shown either equivalent or inferior outcomes in patients randomized to GDHT following major abdominal surgery. Therefore, we performed this up-to-date systematic review and meta-analysis to evaluate all available evidence regarding the effect of preoperative GDHT in comparison with conventional fluid therapy on postoperative recovery in adults undergoing major abdominal surgery.

## Methods

We followed Preferred Reporting Items for Systematic Reviews and Meta-Analyses (PRISMA) guidelines in reporting this systematic review and meta-analysis [15]. A review protocol was written before this study was conducted.

### Inclusion and exclusion criteria

The eligible studies of this systematic review and meta-analyses were identified using the patient, intervention, comparison, outcomes, study design strategy [16]:

1. *Patients/participants*: Adult patients (aged ≥16 years) undergoing major abdominal surgery were evaluated. Major abdominal surgery was defined using the Physiological and Operative Severity Score for the enUmeration of Mortality and Morbidity [17]. Studies involving pediatric patients, nonsurgical patients, or postoperative patients with already-established sepsis or organ failure and undergoing late optimization were excluded.
2. *Type of intervention*: Preoperative GDHT was used as the intervention treatment, which was defined as preoperative administration of fluids (initiated before surgery or in the intraoperative period and maintained in the postoperative period, or performed in the immediate postoperative period and lasting up to 6 h after surgery), with or without inotropes/vasoactive drugs, to increase blood flow (relative to control) against explicit measured goals, defined as cardiac output (CO), cardiac index, oxygen delivery (DO_2_), oxygen delivery index (DO_2_I), oxygen consumption, stroke volume (SV), dynamic measures of preload responsiveness (e.g., stroke volume variation [SVV], pulse pressure variation [PPV], and pleth variability index [PVI]), mixed venous oxygen saturation, oxygen extraction ratio, or lactate. Studies in which GDHT was limited to the preoperative period were excluded.
3. *Type of comparator*: Conventional fluid administration strategies were used as control group, defined as that using the standard monitoring parameters (BP, HR, urine output, and CVP) to guide fluid therapy.
4. *Types of outcomes*: Studies in which researchers reported postoperative complications, mortality, and GI function recovery outcomes (i.e., time to tolerate oral diet, time to first flatus, and time to first bowel movement) were included.
5. *Types of studies*: Randomized controlled trials (RCTs), with or without blinding, were included. Data derived from letters, case reports, reviews, or cohort studies were excluded.

### Search strategy and study selection

A systematic search of MEDLINE, Embase, CINAHL, Scopus, the Cochrane Controlled Trials Register, and Cochrane Database of Systematic Reviews from inception to November 2016 was performed to identify relevant studies using the following search terms: “surgery,” “fluid,” “goal directed,” “end point,” “hemodynamic,” “target,” “goal,” and “randomized controlled trials.” Detailed search information used in MEDLINE is presented in Appendix 1. No language restriction was placed on our search. Ongoing trials were searched in the ClinicalTrials.gov databased as well as in conference abstracts, which might provide results even though the trials have not been published yet. Furthermore, the reference lists of the identified reports, reviews, and other relevant publications were reviewed to find additional relevant trials. The reference lists of all eligible publications and reviews were scanned to identify additional studies. Two authors (YS and FC) independently screened and reviewed all titles and abstracts for eligibility. For abstracts that did not provide sufficient information to determine eligibility, full-length articles were retrieved. Agreement between the two authors for inclusion of screened articles was measured using weighted kappa, and disagreement on inclusion or exclusion of articles was resolved by consensus.

### Data extraction

Studies were reviewed and data were extracted independently by two authors (YS and FC) using a predesigned standard form, with any discrepancy being resolved by reinspection of the original article. The following data points were extracted: first author, year of publication, total number of patients, patients’ characteristics, abdominal procedures, the GDHT strategy (goals, monitoring methods, and interventions). The primary endpoints of this review included long-term mortality (i.e., death in longest available follow-up), short-term mortality (i.e., death in the hospital or within 30 days after surgery), and overall complication rates (i.e., number of patients with complications after surgery). The secondary outcome was recovery of GI function, including time to toleration of an oral diet, time to first flatus, and time to first bowel movement. Authors were contacted for missing information about fluid management or data on postoperative recovery. If detailed information was not received, data from such studies were excluded from the present meta-analysis.

### Risk-of-bias assessment

The Cochrane Collaboration’s tool [18] for assessing risk of bias was applied independently by two authors. Risk of bias was assessed as high, low, or unclear for each of selection bias, performance bias, detection bias, attrition bias, and reporting bias. Information for judging the risk of bias was collected from all reports originating from one study, as well as from the protocol published in the registry, if applicable. Appropriate allocation to group assignment and concealment of randomization were considered more important than other domains for minimizing risk of bias in evaluating the effect of GDHT on postoperative recovery after major abdominal surgery, and the reviewers gave more importance to these domains when deciding on overall risk of bias. Agreement between the two reviewers on overall risk-of-bias assessment was determined using weighted kappa as well. Disagreements were resolved through discussion.

### Grading quality of evidence

The quality of evidence for each outcome was assessed according to Grading of Recommendations, Assessment, Development and Evaluations (GRADE) methods for risk of bias, inconsistency, indirectness, imprecision, and publication bias, and it was evaluated using GRADEPro software 3.6 (GRADE Working Group). These were classified as very low, low, moderate, or high [19, 20].

### Statistical analysis

All statistical analyses were conducted using RevMan 5.1 (The Cochrane Collaboration, Oxford, UK) and Stata/SE software 10.0 (StataCorp, College Station, TX, USA). Meta-analysis was undertaken where data were sufficient. For continuous data, weighted mean differences (WMDs) with 95% CIs were calculated. If the 95% CI included 0, the difference between the GDHT and control groups was not considered statistically significant. When mean and SD values were not given, they were estimated from the median and SE or CI or from the IQR using the method described by Hozo et al. [21]. Dichotomous data were analyzed by use of risk ratio (RR) with 95% CI. If the 95% CI around the RR did not include 1.0, the difference between the GDHT and control groups was assumed to be statistically significant. We assessed the included studies for functional equivalence, but we additionally used the Cochran chi-square *Q* and *I* ^2^ statistics to assess heterogeneity across studies. Heterogeneity was considered as either a *p* value <0.05 or *I* ^2^ > 25% [22]. The use of either a fixed-effect or random-effect model was based on a combination of these methods.

The univariate meta-regression analyses were conducted when appropriate (i.e., number of studies >10) to explore the potential heterogeneity according to type of monitoring technology, type of interventions, therapeutic goals, whether in context with enhanced recovery programs, and overall “fitness” of the patients (i.e., high- risk patients versus non-high-risk patients). High-risk patients were defined as patients with an American Society of Anesthesiologists physical status classification of III with two or more risk factors according to the risk index of Lee (i.e., high-risk type of surgery, ischemic heart disease, history of congestive heart failure, history of cerebrovascular disease, insulin therapy for diabetes, and preoperative serum creatinine >2.0 mg/dl) [7]. Moreover, prespecified subgroup analyses were conducted on the basis of these potential confounders to minimize heterogeneity and evaluate the effect of GDHT in the specific subpopulations. Additional sensitivity analyses were performed, including studies for colorectal surgical procedures, studies randomizing large-sample-size patients (defined as sample size ≥100), and studies judged to carry a low risk of bias. Finally, the influence of each study was evaluated on the basis of overall estimates by calculating random-effect pooled estimates, omitting each estimate one at a time [23].

Publication bias was evaluated using Begg’s funnel plots. Two formal tests—Begg’s adjusted rank correlation and Egger’s regression asymmetry test—were also used to assess publication bias [24, 25].

## Results

**Fig. 1:**
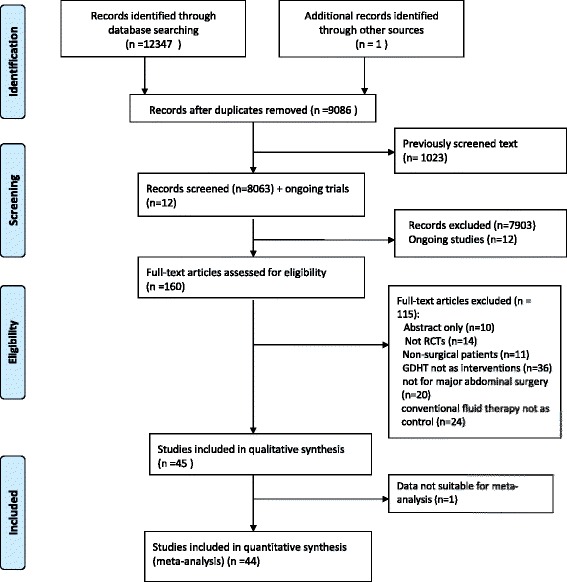
Preferred Reporting Items for Systematic Reviews and Meta-Analyses (PRISMA) diagram of study selection. *GDHT* Goal-directed hemodynamic therapy, *RCT* Randomized controlled trial

There were 12,348 records for title and abstract screening. After applying inclusion and exclusion criteria, 12,188 citations were excluded because of duplication of published data, not reporting original research, or no human patients being involved. The remaining subset of 160 articles was gathered for further review. This group was evaluated in detail by each author to reach consensus on whether the articles met the inclusion criteria described above until full consensus was reached. Of this group, 115 articles were excluded because they were not RCTs, involved nonsurgical patients, did not evaluate the effect of GDHT, did not involve major abdominal surgery, did not use conventional fluid therapy as a control group, or were published only in letter or abstract form. A total of 45 RCTs were finally considered for this review (Fig. 1). The authors had perfect agreement in selecting the 45 studies using the stated eligibility criteria.

### Study characteristics

The 45 RCTs [1, 11–14, 26–65] yielded 6344 patients (Table 1). Of those patients, 3406 received perioperative GDHT. Sample sizes ranged from 27 to 1994. All studies were reported between 1988 and 2015 in English-language journals.

**Table 1:** Study characteristics and overall risk of bias assessment for each study

Trial/author, year [reference]	Number of patients	Nature of surgery	Goal-directed hemodynamic therapy			ERP performed	Overall risk of bias
			Goal	Monitoring method	Interventions		
Bender et al., 1997 [26]	106	Elective infrarenal aortic surgery or lower limb revascularization	Cardiac index ≥2.8 L/minute/m^2^, SVR ≤1100 dyn/second/cm^5^, PAWP 8–14 mmHg	PAC	Fluids and inotropes	No	High
Benes et al., 2010 [27]	120 (high-risk)	Open major abdominal surgery	SVV 10%, cardiac index 2.5–4.1 L/minute/m^2^, and MAP >70 mmHg	Pulse contour analysis monitor	Fluids, inotropes, and vasopressors	No	Low
Bisgaard et al., 2013 [28]	64	Open abdominal aortic surgery	Optimal SV, DO_2_I >600 ml/minute/m^2^, HR <100 beats/minute	Pulse contour analysis monitor	Fluids and inotropes	No	Low
Bonazzi et al., 2002 [29]	100	Open abdominal aortic surgery	SVR ≤1450 dyn/second/cm^5^, PAOP 10–18 mmHg, cardiac index >3 L/minute/m^2^, and DO_2_I >600 ml/minute/m^2^	PAC	Fluids and inotropes	No	Unclear
Brandstrup et al., 2012 [11]	150	Open and laparoscopic colorectal surgery	SV 10%	Esophageal Doppler	Fluids	Yes	Low
Buettner et al., 2008 [31]	80	Major abdominal surgery	SPV <10%	Pulse contour analysis monitor	Fluids	No	Low
Boyd et al., 1993 [30]	107 (high-risk)	Major abdominal surgery	DO_2_I >600 ml/minute/m^2^	PAC	Fluids and inotropes	No	Unclear
Bundgaard-Nielsen et al., 2013 [63]	42	Open radical prostatectomy	Optimal SV	Esophageal Doppler	Fluids	Yes	Low
Cohn et al., 2010 [47]	27	Open colorectal surgery	StO_2_ > 75%	Near-infrared spectroscopy	Fluids	No	Low
Correa-Gallego et al., 2015 [12]	135	Liver resection	SVV baseline	Pulse contour analysis monitor	Fluids and inotropes	No	Low
Challand et al., 2011 [32]	179 (56 high-risk)	Major open or laparoscopic colorectal surgery	SV 10%	Esophageal Doppler	Fluids	Yes	Low
Conway et al., 2002 [33]	57	Colorectal resection	SV 10%, FTc >0.35	Esophageal Doppler	Fluids	No	Unclear
Donati et al., 2007 [34]	135 (high-risk)	Major abdominal surgery	O_2_ER ≤27%	Central line + arterial line sampling	Fluids and inotropes	No	Unclear
Forget et al., 2010 [35]	82	Major abdominal surgery	Pleth variability index <13%	Pulse oximeter	Fluids	No	Unclear
Gan et al., 2002 [36]	100 (high-risk)	Major open abdominal surgery	SV 10%, FTc >0.35	Esophageal Doppler	Fluids	No	Low
Jammer et al., 2010 [37]	241	Open colorectal surgery	Central venous oxygen saturation >75%	Central line	Fluids	No	Low
Jhanji et al., 2010 [38]	135	Major gastrointestinal surgery	Optimal SV	Pulse contour analysis monitor	Fluids or fluids and inotropes	No	Low
Jones et al., 2013 [39]	91	Liver resection	Optimal SV	Pulse contour analysis monitor	Fluids	Yes^a^	Unclear
Lopes et al., 2007 [40]	33	Major abdominal surgery	Variation in arterial pulse pressure <10%	Arterial line + monitoring	Fluids and inotropes	No	Low
Mayer et al., 2010 [41]	60 (high-risk)	Major abdominal surgery	SVV 12%, cardiac index ≥2.5 L/minute/m^2^, SVI >35 ml/m^2^	Pulse contour analysis monitor	Fluids, inotropes, and vasopressors	No	Unclear
McKenny et al., 2013 [62]	101	Major gynecologic surgery	Optimal SV	Esophageal Doppler	Fluids	No	Low
Noblett et al., 2006 [42]	103	Open and laparoscopic colorectal surgery	FTc >0.35, SV	Esophageal Doppler	Fluids	Yes	Unclear
Pearse et al., 2014 [1]	734 (high-risk)	Major abdominal surgery	Optimal SV	Pulse contour analysis monitor	Fluids and inotropes	No	Low
Pearse et al. 2005 [43]	122 (high-risk)	General, vascular, and urologic surgery	DO_2_I >600 ml/minute/m^2^	Pulse contour analysis monitor	Fluids and inotropes	No	Low
Pestana et al., 2014 [13]	142	Major gastrointestinal surgery	Cardiac index ≥2.5 ml/minute/m^2^, MAP >65 mmHg	Noninvasive cardiac output monitor	Fluids, vasopressors, and inotropes	No	Low
Phan et al., 2014 [14]	100	Colorectal surgery	FTc >0.35, SV	Esophageal Doppler	Fluids	Yes	Unclear
Phillai et al., 2011 [44]	66	Radical cystectomy for bladder cancer	FTc >0.35, SV	Esophageal Doppler	Fluids	Yes	Unclear
Ramsingh et al., 2013 [45]	38 (high-risk)	Open major abdominal surgery	SVV <12%	Pulse contour analysis monitor	Fluids and inotropes	No	Low
Salzwedel et al., 2013 [61]	160	Major abdominal surgery	PPV 10%, cardiac index >2.5 ml/minute/m^2^	Pulse contour analysis monitor	Fluids and inotropes, vasopressors	No	Low
Sandham et al., 2003 [46]	1994 (high-risk)	Major abdominal, thoracic, vascular, or hip fracture surgery	DO_2_I 550–600 ml/minute/m^2^, cardiac index 3.5–4.5 ml/minute/m^2^, MAP >70 mmHg, PAOP 18 mmHg, HR <120 beats/minute, Hct >27%	PAC	Fluids and inotropes	No	Low
Scheeren et al., 2013 [65]	52 (high-risk)	Major abdominal surgery, radical cystectomy	SVV 10%	Pulse contour analysis monitor	Fluids and inotropes	No	Low
Senagore et al., 2009 [48]	64	Laparoscopic colectomy	FTc >0.35, SV	Esophageal Doppler	Fluids	Yes	Unclear
Shoemaker et al., 1988 [50]	88 (high-risk)	Major abdominal surgery and other types of surgery	CO >4.5 L/minute, DO_2_I >600 mL/minute/m^2^, VO_2_ > 170 ml/minute/m^2^	PAC	Fluids and inotropes	No	Low
Sharkawy et al., 2013 [49]	59	Major liver resection	FTc >0.35, SV 10%	Esophageal Doppler	Fluids	No	Unclear
Srinivasa et al., 2012 [51]	85	Open or laparoscopic colectomy	FTc >0.35, SV	Esophageal Doppler	Fluids	Yes	Low
Szakmany et al., 2005 [52]	45	Major abdominal surgery	ITBVI 850–950 ml/m^2^	Pulse contour analysis monitor	Fluids	No	Unclear
Ueno et al., 1997 [53]	34	Major liver resection	DO_2_I >600 ml/minute/m^2^, cardiac index >4.5 L/minute/m^2^, VO_2_ > 170 ml/minute/m^2^	PAC	Fluids and inotropes	No	High
Valentine et al., 1998 [54]	120	Abdominal aortic surgery	Cardiac index ≥2.8 L/minute/m^2^, PCWP 8–15 mmHg, SVR ≤1100 dyn/second/cm^5^	PAC	Fluids and inotropes	No	High
Wilson et al., 1999 [56]	138 (high-risk)	General surgery, vascular surgery, and urologic surgery	DO_2_I >600 ml/minute/m^2^, PAOP 12 mmHg, Hb >110 g/L, SaO_2_ > 94%	PAC	Fluids and inotropes	No	Unclear
Wakeling et al., 2005 [55]	128	Colorectal resection	SV 10%, CVP did not rise by 10mmHg or more	Esophageal Doppler	Fluids	Yes	Unclear
Yu et al., 2010 [57]	299	Gastrointestinal surgery	Central venous lactate <1.6 mmol/L	Central line sampling	Fluids and inotropes	Yes	Low
Zakhaleva et al., 2013 [64]	74	Major abdominal surgery	FTc >0.35, SV 10%	Esophageal Doppler	Fluids	Yes	Low
Zeng et al., 2014 [60]	60	Abdominal cancer surgery	SVV	Pulse contour analysis monitor	Fluids and inotropes	No	Unclear
Zhang et al., 2012 [58]	60	Open gastrointestinal surgery	Pulse pressure variation	Pulse oximeter	Fluids	No	Low
Zheng et al., 2013 [59]	60 (high-risk)	Open gastrointestinal surgery	Cardiac index ≥2.5 L/minute/m^2^, SVI >35 ml/m^2^, MAP >65 mmHg	Pulse contour analysis monitor	Fluids and inotropes	Yes	Low

*Abbreviations: BP* Blood pressure, *CO* Cardiac output, *CVP* Central venous pressure, *DO*_*2*_ *I* Oxygen delivery index, *ERP* Enhanced recovery protocol, *FTc* Corrected flow time, *Hct* Hematocrit, *Hb* Hemoglobin, *HR* Heart rate, *ITBVI* Intrathoracic blood volume index, *MAP* Mean arterial pressure, *O*_*2*_ *ER* Oxygen extraction ratio, *PAC* Pulmonary arterial catheter, *PAOP* Pulmonary arterial occlusion pressure, *PAWP* Pulmonary arterial wedge pressure, *PCWP* Pulmonary capillary wedge pressure, *SPV* Systolic pressure variation, *StO*_*2*_ Tissue blood oxygen saturation, *SV* Stroke volume, *SVI* Stroke volume index, *SVV* Stroke volume variation, *SVR* Systemic vascular resistance, *VO*_*2*_ Oxygen consumption

^a^ ERP performed only in study group

**Fig. 2:**
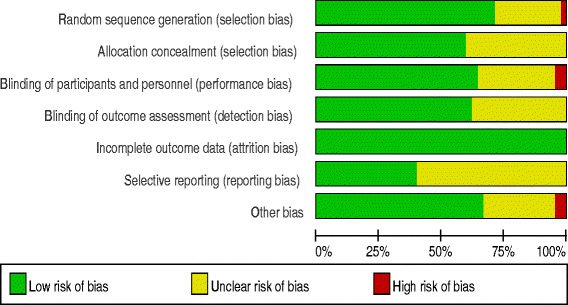
Review authors’ judgments about each risk of bias item presented as percentages across all included studies

Bias risk was analyzed with the Cochrane tool. The methodological quality of included trials is presented in a summary graph (Fig. 2) and table (Additional file 1). A total of 26 studies (58%) [1, 3, 11–13, 27, 28, 31, 32, 36–38, 40, 43, 45–47, 50, 51, 57–59, 61, 62, 64, 65] were judged to carry a low risk of bias (Table 1). Weighted kappa was calculated to examine agreement for each component and overall risk of bias assessment. The kappa statistics showed substantial agreement between the reviewers (Additional file 2).

Eight trials [26, 29, 30, 46, 50, 53, 54, 56] used pulmonary arterial catheters for monitoring; fourteen trials [3, 11, 14, 32, 33, 36, 42, 44, 48, 49, 51, 55, 62, 64] used esophageal Doppler monitoring; fifteen trials [3, 12, 27, 28, 31, 38, 39, 41, 43, 45, 52, 59–61, 65] used self-calibrating/calibrated pulse contour analysis monitoring; and the remaining eight trials used other monitors, including arterial lines plus monitoring equipment [40], central lines and arterial line sampling [34, 37, 57], pulse oximeters [35, 58], and other noninvasive monitors [13, 47]. Three types of goals were used in the majority of included trials, including DO_2_I and/or cardiac index [13, 26, 29, 30, 43, 46, 50, 53, 54, 56, 59], optimal SV [1, 11, 28, 32, 33, 36, 38, 39, 42, 44, 48, 49, 51, 55], and dynamic measures of preload responsiveness (e.g., PPV, SVV, PVI) [12, 27, 31, 35, 40, 41, 45, 52, 58, 60, 61, 65].

### Meta-analyses

#### Long-term mortality

**Fig. 3:**
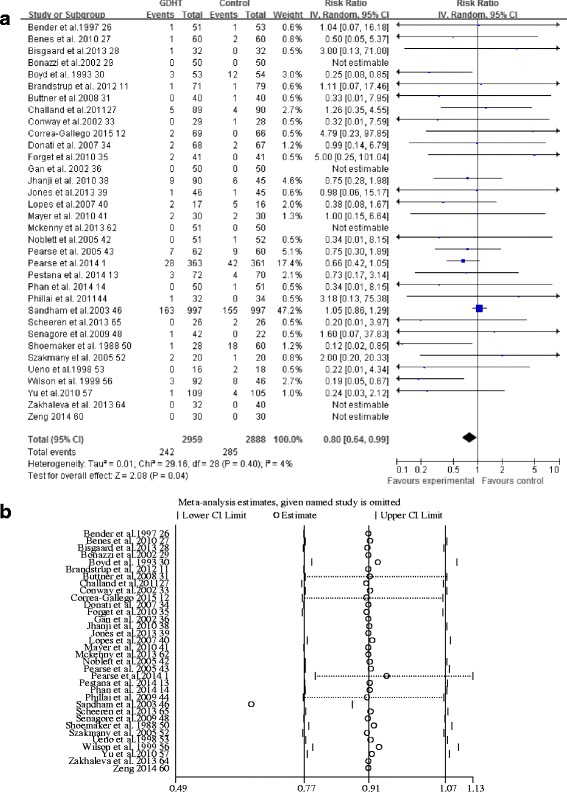
Meta-analysis and pooled risk ratio (RR) of the effect of perioperative goal-directed hemodynamic therapy (GDHT) on long-term mortality after major abdominal surgery and the influence analysis of individual studies on the pooled RR. Forest plots for (**a**) long-term mortality and (**b**) the influence of individual studies on the pooled RR

Thirty-three trials [1, 11, 14, 26–36, 38–43, 46, 48, 50, 52, 53, 56, 57, 60, 62, 64, 65] provided data on long-term mortality, and further information was obtained from a previous meta-analysis [8] for one study [44]. The long-term mortality was 242 (8.1%) of 2959 in the GDHT group and 285 (9.9%) of 2888 in the control group, and the pooled RR of 0.80 showed that use of perioperative GDHT was barely associated with improved long-term survival after major abdominal surgery compared with the control group (95% CI 0.64–99, *p* = 0.04; *I* ^2^ = 4%) (Fig. 3a). The GRADE quality of evidence was judged to be moderate, downgraded for risk of bias.

Subgroup analyses revealed that a statistically significant effect of GDHT in long-term mortality for high-risk patients (RR 0.57, 95% CI 0.36–0.89, *p* = 0.01; *I* ^2^ = 51%; number of studies [*n*] = 12 [1, 27, 30, 32, 34, 36, 41, 43, 46, 50, 56, 65]), patients using cardiac index and/or DO_2_I as therapeutic goals (RR 0.48, 95% CI 0.25–0.94, *p* = 0.03; *I* ^2^ = 60%; *n* = 9 [13, 26, 29, 30, 43, 46, 50, 53, 56]), and patients using fluids and inotropes as interventions (RR 0.63, 95% CI 0.44–0.89, *p* = 0.008; *I* ^2^ = 32%; *n* =20 [1, 12, 13, 26–30, 34, 38, 40, 41, 43, 46, 50, 53, 56, 57, 60, 65]) (Additional file 3). Meta-regression analyses did not find the significant effect of overall “fitness” of the patients, type of monitoring technology, type of intervention, therapeutic goals, and whether in context with enhanced recovery programs on our result (Additional file 4). No statistical difference was found when we analyzed studies for colorectal surgical procedures, studies randomizing large-sample-size patients, and studies carrying a low risk of bias (Additional file 3). The influence analyses showed that each study except one trial [46] had a minor influence on the overall pooled RR. The statistical difference between the GDHT and control groups reached significance after this trial was omitted (RR 0.63, 95% CI 0.48–0.83, *p* = 0.001) (Fig. 3b). Neither Begg’s adjusted rank correlation test (*p* = 0.10) nor Egger’s regression asymmetry test (*p* = 0.93) was significant for mortality. A funnel plot is presented in Additional file 5.

#### Short-term mortality

**Fig. 4:**
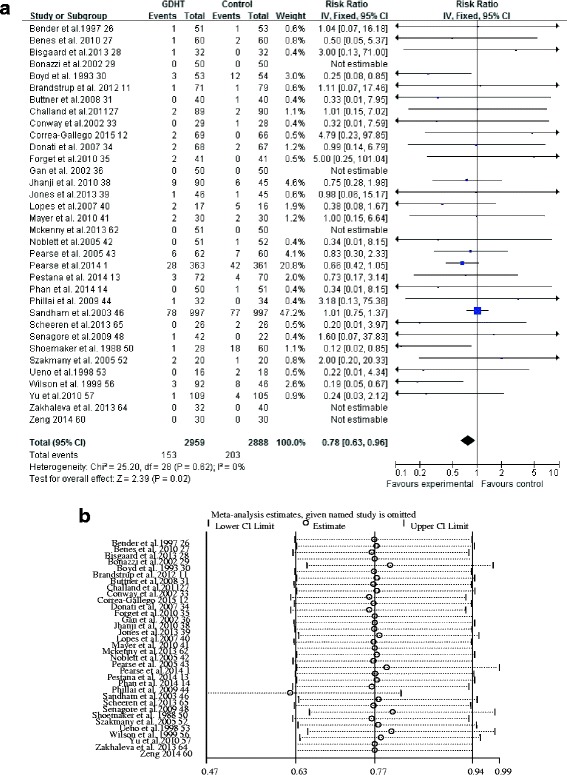
Meta-analysis and pooled risk ratio (RR) of the effect of perioperative goal-directed hemodynamic therapy (GDHT) on short-term mortality after major abdominal surgery and the influence analysis of individual studies on the pooled RR. Forest plots for (**a**) short-term mortality and (**b**) the influence of individual studies on the pooled RR

Thirty-four studies [1, 11–14, 26–36, 38–41, 43, 44, 46–48, 50, 52–54, 56, 60, 62, 64, 65] provided suitable data for analysis. The pooled short-term mortality was 153 (5.2%) of 2959 in the GDHT group and 203 (7.0%) of 2888 in the control group, and the RR was 0.75 (95% CI 0.61–0.91, *p* = 0.004; *I* ^2^ = 0%), showing a significant reduction in the GDHT group (Fig. 4a). The GRADE quality of evidence was judged to be moderate, downgraded for risk of bias.

In subgroup analyses, we found that GDHT significantly reduced short-term mortality when a pulmonary arterial catheter was used for monitoring (RR 0.36, 95% CI 0.14–0.96, *p* = 0.04; *I* ^2^ = 68%; *n* = 7 [26, 29, 30, 46, 50, 53, 56]), cardiac index and/or DO_2_I were used as therapeutic goals 2(RR 0.49, 95% CI 0.25–0.94, *p* = 0.03; *I* ^2^ = 55%; *n* = 9 [13, 26, 29, 30, 43, 46, 50, 53, 56]), fluids and inotropes were used as interventions (RR 0.65, 95% CI 0.47–0.89, *p* = 0.007; *I* ^2^ =19%; *n* = 20 [1, 12, 13, 26–30, 34, 38, 40, 41, 43, 46, 50, 53, 56, 57, 60, 65]), outside of enhanced recovery programs (RR 0.71, 95% CI 0.53–0.94, *p* < 0.0001; *I* ^2^ = 11%; *n* = 25 [1, 12, 26–28, 31, 33–35, 38, 40, 41, 43]), and for high-risk patients (RR 0.73, 95% CI 0.58–0.91, *p* = 0.09; *I* ^2^ = 39%; *n* = 12 [1, 27, 30, 32, 34, 36, 41, 43, 46, 50, 56, 65]) (Additional file 3). Again, meta-regression analysis failed to identify the significant factors contributing this result (Additional file 6). No statistical difference was found when we analyzed studies for colorectal surgical procedures, studies randomizing large-sample-size patients, and studies carrying a low risk of bias (Additional file 3). The influence analyses showed each study had no substantial influence on the overall pooled RR (Fig. 4b). A funnel plot is presented in Additional file 7. Neither Begg’s adjusted rank correlation test (*p* = 0.08) nor Egger’s regression asymmetry test (*p* = 0.48) showed evidence of publication bias regarding short-term mortality.

#### Overall complication rates

**Fig. 5:**
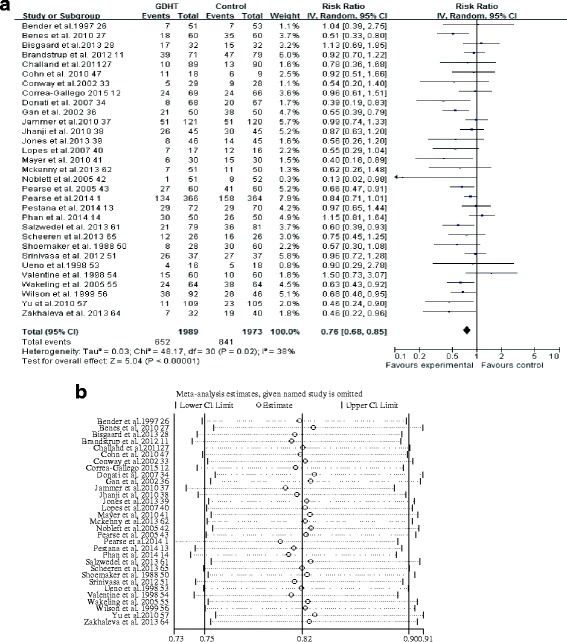
Meta-analysis and pooled risk ratio (RR) of the effect of perioperative goal-directed hemodynamic therapy (GDHT) on overall complication rates after major abdominal surgery and the influence analysis of individual studies on the pooled RR. Forest plots for (**a**) overall complication rates and (**b**) the influence of individual studies on the pooled RR

Thirty-one trials [11–13, 26–28, 32–34, 36–39, 41–43, 47, 49, 50, 53–57, 61, 62, 64, 65] reported suitable data on number of patients with complications. The pooled RR of 0.76 showed reduced overall complication rates after major abdominal surgery in the GDHT group compared with the control group (95% CI 0.68–0.85, *p* < 0.0001; *I* ^2^ = 38%) (Fig. 5a). The GRADE quality of evidence was judged to be low, downgraded for risk of bias and inconsistency.

Subgroup analyses showed a significant reduction in GDHT group in those studies using pulse contour analysis monitoring (RR 0.75, 95% CI 0.64–0.87, *p* = 0.003; *I* ^2^ = 33%; *n* = 10 [1, 12, 27, 28, 38, 39, 41, 43, 61, 65]), using esophageal Doppler monitoring (RR 0.75, 95% CI 0.58–0.95, *p* = 0.002; *I* ^2^ = 53%; *n* = 10 [11, 14, 32, 33, 36, 42, 51, 55, 62, 64]), using fluids and inotropes as interventions (RR 0.76, 95% CI 0.66–0.86, *p* < 0.0001; *I* ^2^ = 36%; *n* = 19 [1, 12, 13, 26–28, 34, 38, 40, 41, 43, 50]), using cardiac index and/or DO_2_I (RR 0.78, 95% CI 0.63–0.97, *p* = 0.03; *I* ^2^ = 18%; *n* = 7 [1, 26, 43, 53, 54, 56]), or optimal SV (RR 0.80, 95% CI 0.69–0.93, *p* = 0.0002; *I* ^2^ = 40%; *n* = 14 [1, 10, 11, 14, 28, 32, 33, 36, 38, 39, 42, 55, 62, 64]) or dynamic measures of preload responsiveness (RR 0.64, 95% CI 0.52–0.79, *p* < 0.0001; *I* ^2^ = 16%; *n* = 6 [12, 27, 40, 41, 61, 65]) as therapeutic goals, as well as for either high-risk patients (RR 0.65, 95% CI 0.56–0.76, *p* < 0.0001, *I* ^2^ = 28%; *n* = 10 [1, 27, 32, 34, 36, 41, 43, 50, 56, 65]) or non-high-risk patients (RR 0.84, 95% CI 0.74–0.96, *p* = 0.08; *I* ^2^ = 29%; *n* = 21 [11–14, 26, 28, 33, 37–40, 42, 47, 51, 53–55, 57, 61, 62, 64]) (Additional file 3). Meta-regression analyses did not reveal a significant effect of all predefined confounders on overall complication rates (Additional file 8). Additionally, a statistically significant effect of GDHT on overall complication rates was found when we pooled all studies carrying to a low risk of bias (RR 0.78, 95% CI 0.70–0.87, *p* < 0.0001; *I* ^2^ = 31% ; *n* = 20 [1, 11–13, 27, 28, 32, 36–38, 40, 43, 47, 50, 51, 57, 61, 62, 64, 65]) and studies randomizing large-sample-size patients (RR 0.79, 95% CI 0.69–0.89, *p* = 0.002; *I* ^2^ = 43% ; *n* = 19 [1, 11–14, 26, 27, 32, 34, 36–38, 42, 43, 54–57, 62]) (Additional file 3). The influence analyses showed each study had no substantial influence on the overall pooled RR (Fig. 5b). Begg’s test and Egger’s test excluded the presence of publication bias (*p* = 0.08 and *p* = 0.06, respectively). A funnel plot is presented in Additional file 9.

#### GI function recovery

**Fig. 6:**
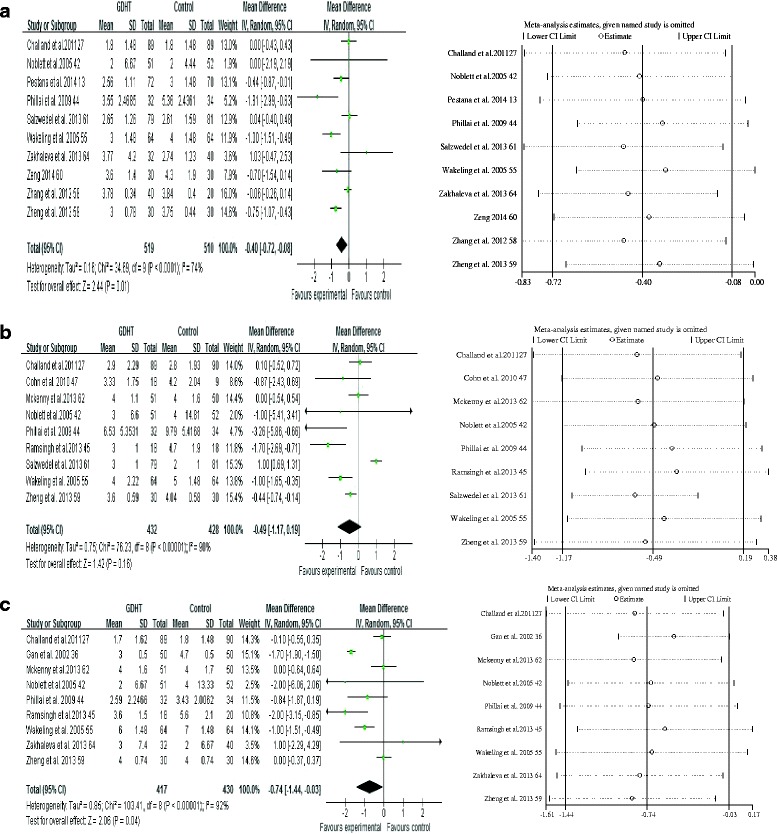
Meta-analysis and pooled weighted mean differences (WMDs) of the effect of perioperative goal-directed hemodynamic therapy (GDHT) on (**a**) time to first flatus pass, (**b**) time to first bowel movement, and (**c**) time to toleration of an oral diet after major abdominal surgery and the influence analysis of individual studies on the WMD. *Left side* shows Forest plots, and *right side* shows the influence of individual studies on the pooled estimates

Perioperative GDHT shortened the time to first flatus (WMD −0.40 days, 95% CI −0.72 to −0.08, *p* < 0.0001; *I* ^2^ = 74%; *n* = 10 [13, 32, 42, 44, 55, 58–61, 64]) and time to toleration of an oral diet (WMD −0.74 days, 95% CI −1.44 to −0.03, *p* < 0.0001; *I* ^2^ = 92%; *n* = 9 [32, 36, 42, 44, 45, 55, 59, 62, 64]), but it did not shorten the time to first bowel movement (Fig. 6). The GRADE quality of evidence was judged to be low, downgraded by risk of bias and inconsistency.

Subgroup analyses based on the type of monitoring and therapeutic goals were not performed, owing to the limited number of studies. A statistically significant effect of GDHT was observed on time to toleration of an oral diet when we pooled studies for non-high-risk patients (WMD −0.83 days, 95% CI −1.51 to −0.14, *p* = 0.03; *I* ^2^ = 59%; *n* = 6 [42, 44, 45, 55, 62, 64]) and on time to first flatus pass for non-high-risk patients (WMD −0.41 days, 95% CI −0.80 to −0.01, *p* = 0.04; *I* ^2^ = 71%; *n* = 8 [13, 42, 44, 55, 58, 60, 61, 64]) and patients using fluids and inotropes as interventions (WMD −0.45 days, 95% CI −0.83 to −0.06, *p* < 0.0001; *I* ^2^ = 64%; *n* = 4 [13, 59–61]). No significant difference between the GDHT and control groups was found by sensitivity analysis restricting studies for colorectal surgical procedures, studies randomizing large-sample-size patients, and studies carrying a low risk of bias. The influence analyses showed that each study had no substantial influence on the overall pooled estimates, except for one trial regarding the time to first bowel movement. After we omitted this study, the difference in the time to first bowel movement reached statistical significance (WMD −0.28 days, 95% CI −0.43 to −0.13, *p* = 0.01) (Fig. 6).

Begg’s test and Egger’s test revealed no evidence of publication bias regarding time to first flatus (*p* = 1.00 and *p* = 0.48, respectively), time to first bowel movement (*p* = 0.91 and *p* = 0.19, respectively), or time to toleration of an oral diet (*p* = 0.28 and *p* = 0.46, respectively). A funnel plot is presented in Additional file 10.

## Discussion

In this systematic review and meta-analysis, we found that perioperative GDHT improved survival, reduced overall complication rates, and facilitated GI functional recovery as demonstrated by shortening the time to first flatus pass and the time to toleration of an oral diet compared with conventional fluid therapy when all studies were considered. However, we did not identify the beneficial effects of GDHT on mortality and GI function when we restricted the analysis to higher-quality and large-sample-size studies; thus, future studies should be adequately powered and methodologically rigorous enough to confirm a clinically relevant effect in this area.

GDHT is currently recommended in the context of enhanced recovery programs, especially for moderate- to high-risk patients [7]. High-risk patients tend to have an increased stress response to surgical aggression, increased oxygen demand, and reduced physiological reserves to deal with the metabolic requirements of the perioperative period. Strategies to maintain DO_2_ and minimize splanchnic hypoperfusion have been advocated to improve postoperative morbidity for high-risk surgical patients [66]. In our subgroup analyses, we identified high-risk patients as a group that may potentially benefit from GDHT. However, the results of our subgroup analysis indicated that GDHT is beneficial mainly when used outside enhanced recovery programs. The potential explanation is that enhanced recovery programs emphasize the avoidance of bowel preparation, minimize fasting, and use preoperative carbohydrate loading [67]. As a result, patients are less likely to be fluid-depleted during surgery and thus may not benefit as much from targeted fluid administration.

Many different GDHT strategies have been studied in the clinical setting. However, there is no clear consensus about the most effective or the most appropriate method of monitoring. One would suggest that the use of CO monitoring to guide administration of intravenous fluids coupled with inotropic drugs as part of a hemodynamic therapy algorithm, which has been shown to modify inflammatory pathways and improve tissue perfusion and oxygenation [68]. In our subgroup analysis, we found that GDHT using cardiac index/DO_2_I as goals and using fluids and inotropes as interventions was associated with reductions in mortality and morbidity following major abdominal surgery. However, the meta-regression analyses did not reveal any significant effect of those confounders contributing to overall results regarding mortality and morbidity after major abdominal surgery. Therefore, future studies are needed to provide evidence supporting various goals and methods of monitoring.

With a number of recently published trials on this topic, this report is the most up-to-date analysis of the effects of GDHT on recovery after major abdominal surgery and is based on a comprehensive search strategy. This systematic review included eight high-quality studies [28, 37, 38, 43, 46, 50, 57, 58] that were not identified in the most recently published meta-analysis [69], as well as two newly published studies [1, 12]. Moreover, we also included 12 studies [26, 30, 34, 39, 41, 47–49, 52–54, 56] that were excluded from the previous meta-analyses. Our findings support results of previous meta-analyses either for all types of surgery [8] or following major abdominal surgery [70].

There are some notable limitations of this review; therefore, the results should be interpreted with caution. Although our systematic review was focused on major abdominal procedures, owing to the unique nature of physiological change, we tried to attenuate the divergent effects of a heterogeneous population [71]. However, the risk-benefit balance may be varied between the surgical procedures on the basis of the degree and duration of physiological stress. First, the results of sensitivity analysis restricted to studies with colorectal surgical procedures did not show the positive effect of GDHT on mortality, morbidity, and GI function recovery. Second, the GDHT strategy is quite complex and varied between trials, including fluid management, monitoring methods, therapeutic goals, and perioperative care environment. None of the included studies mentioned evaluating the effect of a single, clearly defined intervention, and analyzing data from some of the included trials using potential “nonoptimal” regimens might have impacted the results of our meta-analysis. Although our meta-regression analysis did not reveal a statistically significant influence of those confounders on overall results, the possibility of the regimen of GDHT that may be efficacious for postoperative recovery could not be excluded. Third, the quality of outcome data reported in the included studies was variable. Although the subgroup and sensitivity analyses could reduce the heterogeneity, not all planned subgroup and sensitivity analyses could be performed, owing to insufficient suitable data reported. Thus, the observed statistical heterogeneity in certain analyses could not always be ensured. Moreover, outcome measures were not consistent across all studies, and only relevant data from included trials could be considered for meta-analysis because of the limitation of pooled analysis. Although return of GI function is considered a meaningful outcome following abdominal surgery, only 13 of the 45 included trials provided data on this outcome. In addition, a specific analysis of complications was not performed, owing to the varied definitions between studies. Fourth, about half of the included studies had small sample sizes (<100), which may lack statistical power to detect a clinically important difference in mortality. The sensitivity analysis when we restricted it to studies with higher methodological quality and studies with larger sample size did not confirm the results obtained. Finally, as with any meta-analysis, publication bias could not be excluded. Although Begg’s test and Egger’s test were conducted in this analysis and the results indicated no significant evidence for publication bias for each outcome, absence of significant asymmetry does not mean that publication bias was absent [72].

## Conclusions

This systematic review of available evidence suggests that the use of perioperative GDHT could improve postoperative recovery following major abdominal surgery, as demonstrated by a reduction of postoperative morbidity, improvement of survival, and earlier return of GI function. However, the most effective GDHT strategy remains unclear, and future adequately powered, high-quality RCTs are therefore needed to address this issue.

### Additional files

Additional file 1: Risk of bias summary: review authors’ judgments about each risk-of-bias item for each included study. (PDF 341 kb)

Additional file 2: Weighted kappa measurements to assess agreement between reviewers in rating quality of methodology of included trials. (PDF 48 kb)

Additional file 3: Results of subgroup analysis and sensitivity analyses for mortality and overall complication rates. *RR* Risk ratio, *CI* 95% Confidence interval, *ERP* Enhanced recovery protocol, *N* Number of studies, *n* Number of participants, *PAC* Pulmonary arterial catheter, *OEDM* Esophageal Doppler monitor, *CI#* Cardiac index, *DO*_*2*_ *I* Oxygen delivery index, *SV* Stroke volume, *SVV* Stroke volume variation. (1) Self-calibrating/calibrated pulse contour analysis monitor for example, Vigileo/Flotrac, LiDCO, PiCCO. (2) Arterial line monitoring equipment, central line and arterial line sampling, pulse oximeter, and other noninvasive monitors. (3) Pulse pressure variation (PPV), variation in arterial pulse pressure, and pleth variability index (PVI). (4) Mixed venous oxygen saturation, oxygen extraction ratio, or lactate. * Statistically significant. (DOCX 20 kb)

Additional file 4: Meta-regression analysis for long-term mortality based on type of patients (high-risk versus non-high-risk), type of monitoring used, type of interventions (fluids versus fluids and inotropes), therapeutic goals, and whether in context with enhanced recovery programs (ERPs). *RR* Risk ratio. (PDF 100 kb)

Additional file 5: Begg’s publication funnel plots on long-term mortality. *RR* Risk ratio. (PDF 127 kb)

Additional file 6: Meta-regression analysis for short-term mortality. *ERP* Enhanced recovery program. (PDF 99 kb)

Additional file 7: Publication funnel plots for short-term mortality. *RR* Risk ratio. (PDF 82 kb)

Additional file 8: Meta-regression analysis for overall complication rates. *RR* Risk ratio, *ERP* Enhanced recovery program. (PDF 18 kb)

Additional file 9: Begg’s publication funnel plots on overall complication rates. *RR* Risk ratio. (PDF 128 kb)

Additional file 10: Begg’s publication funnel plots on time to first flatus pass (a), time to bowel movement (**b**), and time to tolerate oral diet (**c**). *WMD* Weighted mean difference. (PDF 54 kb)

## Appendix 1

### MEDLINE search strategy

exp Fluid Therapy/

exp Body Fluids/

exp Echocardiography, Doppler/

exp Echocardiography, Transesophageal/

exp Ultrasonography, Doppler/

exp Cardiac Output/

exp Monitoring, Intraoperative/

exp Blood Flow Velocity/

exp Hemodynamics/

exp Stroke Volume/

exp Blood Pressure/

exp Pulmonary Artery/

exp Catheterization, Swan-Ganz/

exp Thermodilution/

exp Monitoring, Physiologic/

exp Pulse/

exp Intraoperative Care/or exp Intraoperative Period/

exp Oximetry/

Oxygen/or exp Oxygen Consumption/

exp Critical Care/

exp Biological Oxygen Demand Analysis/

exp Vascular Access Devices/

exp Arterial Pressure/

exp Central Venous Catheters/

exp Venous Pressure/

exp Manometry/

exp Models, Cardiovascular/

exp Cardiography, Impedance/

exp Cardiopulmonary Resuscitation/

exp Plethysmography, Impedance/or Plethysmography/

exp Heart Function Tests/

exp Indicator Dilution Techniques/

exp Radioisotope Dilution Technique/

exp Lithium Chloride/

exp Microdialysis/

exp Colloids/

exp Heart Rate/

exp Aorta/

exp Spectrum Analysis/

exp Spectroscopy, Near-Infrared/

exp Electric Impedance/

goal directed therapy.tw.

goal.tw.

exp Carbon Dioxide/

exp Pulsatile Flow/

exp Cardiac Volume/

exp Cardiac Output, Low/

exp Cardiac Output, High/

exp Diagnostic Techniques, Cardiovascular/

exp Plasma Substitutes/

1 or 2 or 3 or 4 or 5 or 6 or 7 or 8 or 9 or 10 or 11 or 12 or 13 or 14 or 15 or 16 or 17 or 18 or 19 or 20 or 21 or 22 or 23 or 24 or 25 or 26 or 27 or 28 or 29 or 30 or 31 or 32 or 33 or 34 or 35 or 36 or 37 or 38 or 39 or 40 or 41 or 42 or 43 or 44 or 45 or 46 or 47 or 48 or 49 or 50

exp Intestinal Mucosa/

exp Gastric Mucosa/

exp Splanchnic Circulation/

exp abdominal aortic surgery/or exp anastomosis, roux-en-y/or exp appendectomy/or exp biliary tract surgical procedures/or exp biliopancreatic diversion/or exp colectomy/or exp cystectomy/or exp endoscopy, digestive system/or exp enterostomy/or exp fundoplication/or exp gastrectomy/or exp gastroenterostomy/or exp gastropexy/or exp gastroplasty/or exp gastrostomy/or exp hemorrhoidectomy/or exp hepatectomy/or exp jejunoileal bypass/or exp liver transplantation/or exp pancreas transplantation/or exp pancreatectomy/or exp pancreaticoduodenectomy/or exp pancreaticojejunostomy/or exp peritoneovenous shunt/

exp Abdomen/

exp Laparoscopy/or exp Hand-Assisted Laparoscopy/

exp Laparotomy/

exp Colostomy/

exp Ileostomy/

exp Colonic Pouches/

exp Proctocolectomy, Restorative/

intermediate risk patients.mp.

high risk patients.mp.

abdominal surgery.mp.

52 or 53 or 54 or 55 or 56 or 57 or 58 or 59 or 60 or 61 or 62 or 63 or 64 or 65

51 and 66

exp Randomized Controlled Trial/

67 and 68

exp = explod

## Abbreviations

BP: Blood pressure
CO: Cardiac output
CVP: Central venous pressure
DO_2_: Oxygen delivery
DO_2_I: Oxygen delivery index
ERP: Enhanced recovery protocol
FTc: Corrected flow time
GDHT: Goal-directed hemodynamic therapy
GI: Gastrointestinal
GRADE: Grading of Recommendations, Assessment, Development and Evaluations
Hb: Hemoglobin
Hct: Hematocrit
HR: Heart rate
ITBVI: Intrathoracic blood volume index
MAP: Mean arterial pressure
O_2_ER: Oxygen extraction ratio
PAC: Pulmonary arterial catheter
PAOP: Pulmonary arterial occlusion pressure
PAWP: Pulmonary arterial wedge pressure
PCWP: Pulmonary capillary wedge pressure
PPV: Pulse pressure variation
PRISMA: Preferred Reporting Items for Systematic Reviews and Meta-Analyses
PVI: Pleth variability index
RCT: Randomized controlled trial
RR: Risk ratio
SPV: Systolic pressure variation
StO_2_: Tissue blood oxygen saturation
SV: Stroke volume
SVI: Stroke volume index
SVR: Systemic vascular resistance
SVV: Stroke volume variation
VO_2_: Oxygen consumption
WMD: Weighted mean difference
