# Supplementary material for: Effect of perioperative goal-directed hemodynamic therapy on postoperative recovery following major abdominal surgery—a systematic review and meta-analysis of randomized controlled trials
Source: Crit Care. 2017 Jun 12;21:141. doi: 10.1186/s13054-017-1728-8 (PMC5467058; doi:10.1186/s13054-017-1728-8)
Supplement: Supplementary file 1 — Risk of bias summary: review authors’ judgments about each risk-of-bias item for each included study. (PDF 341 kb) [file 13054_2017_1728_MOESM1_ESM.pdf]

	Random sequence generation (selection bias)	Allocation concealment (selection bias)	Blinding of participants and personnel (performance bias)	Blinding of outcome assessment (detection bias)	Incomplete outcome data (attrition bias)	Selective reporting (reporting bias)	Other bias
Bender et al.1997 26	-	?	-	?	+	?	-
Benes et al. 2010 27	?	+	+	+	+	?	+
Bisgaard et al.2013 28	+	?	+	+	+	+	+
Bonazzi et al.2002 29	+	?	?	?	+	+	+
Boyd et al. 1993 30	?	?	?	+	+	+	?
Brandstrup et al. 2012 11	+	+	+	?	+	+	+
Bundgaard-Nielsen 2013 63	+	+	?	?	+	+	?
Buttner et al.2008 31	?	+	+	+	+	+	?
Challand et al.201127	?	+	+	+	+	?	+
Cohn et al. 2010 47	+	?	+	+	+	?	+
Conway et al.2002 33	?	?	+	+	+	?	+
Correa-Gallego 2015 12	+	?	+	+	+	+	+
Donati et al. 2007 34	+	+	?	?	+	?	?
Forget et al.2010 35	?	?	+	+	+	?	+
Gan et al. 2002 36	+	+	+	+	+	?	+
Jammer et al.2010 37	+	+	+	+	+	+	+
Jhanji et al. 2010 38	+	+	+	+	+	?	+
Jones et al.2013 39	+	?	?	?	+	+	?
Lopes et al.2007 40	?	+	+	+	+	?	+
Mayer et al. 2010 41	?	+	+	+	+	?	?
Mckenny et al.2013 62	+	+	?	?	+	+	?
Noblett et al.2005 42	?	?	+	?	+	?	+
Pearse et al. 2005 43	+	+	+	+	+	?	+
Pearse et al.2014 1	+	+	+	+	+	+	+
Pestana et al. 2014 13	+	+	+	+	+	+	+
Phan et al. 2014 14	+	?	+	?	+	?	?
Phillai et al. 2009 44	?	?	+	+	+	?	?
Ramsingh et al.2013 45	+	?	+	+	+	+	+
Salzwedel et al. 2013 61	+	+	+	+	+	+	+
Sandham et al.2003 46	+	+	+	+	+	?	+
Scheeren et al.2013 65	+	+	?	+	+	?	+
Senagore et al.2009 48	+	?	+	?	+	?	+
Sharkawy et al. 2013 49	+	+	?	?	+	?	?
Shoemaker et al. 1988 50	+	+	?	+	+	?	?
Srinivasa et al. 2012 51	+	+	+	?	+	+	+
Szakmany et al. 2005 52	+	?	+	?	+	?	+
Ueno et al.1998 53	+	?	?	?	+	?	?
Valentine et al. 1998 54	?	+	-	?	+	?	-
Wakeling et al. 2005 55	?	+	+	?	+	?	+
Wilson et al. 1999 56	+	+	?	+	+	?	?
Yu et al.2010 57	+	+	+	+	+	+	+
Zakhaleva et al. 2013 64	+	?	?	+	+	?	+
Zeng 2014 60	+	?	?	?	+	?	+
Zhang et al. 2012 58	+	+	?	+	+	+	+
Zheng et al. 2013 59	+	+	+	+	+	+	+

Additonal file 1: Risk of bias summary: review authors' judgements about each risk of bias item for each included study
