# Supplementary material for: Effect of perioperative goal-directed hemodynamic therapy on postoperative recovery following major abdominal surgery—a systematic review and meta-analysis of randomized controlled trials
Source: Crit Care. 2017 Jun 12;21:141. doi: 10.1186/s13054-017-1728-8 (PMC5467058; doi:10.1186/s13054-017-1728-8)
Supplement: Supplementary file 2 — Weighted kappa measurements to assess agreement between reviewers in rating quality of methodology of included trials. (PDF 48 kb) [file 13054_2017_1728_MOESM2_ESM.pdf]

	κ
Random sequence generation	1.00
Allocation concealment	0.95
Performance bias	0.90
Detection bias	0.83
Attrition bias	0.92
Reporting bias	0.68
Other bias	0.81
Overall bias	0.89

Additonal file 2: Weighted Kappa Measurements to Assess Agreement Between Reviewers in Rating Quality of Methodology of Included Trials
