# Supplementary material for: Effect of perioperative goal-directed hemodynamic therapy on postoperative recovery following major abdominal surgery—a systematic review and meta-analysis of randomized controlled trials
Source: Crit Care. 2017 Jun 12;21:141. doi: 10.1186/s13054-017-1728-8 (PMC5467058; doi:10.1186/s13054-017-1728-8)
Supplement: Supplementary file 3 — Results of subgroup analysis and sensitivity analyses for mortality and overall complication rates. RR Risk ratio, CI 95% Confidence interval, ERP Enhanced recovery protocol, N Number of studies, n Number of participants, PAC Pulmonary arterial catheter, OEDM Esophageal Doppler monitor, CI# Cardiac index, DO 2 I Oxygen delivery index, SV Stroke volume, SVV Stroke volume variation. (1) Self-calibrating/calibrated pulse contour analysis monitor for example, Vigileo/Flotrac, LiDCO, PiCCO. (2) Arterial line monitoring equipment, central line and arterial line sampling, pulse oximeter, and other noninvasive monitors. (3) Pulse pressure variation (PPV), variation in arterial pulse pressure, and pleth variability index (PVI). (4) Mixed venous oxygen saturation, oxygen extraction ratio, or lactate. * Statistically significant. (DOCX 20 kb) [file 13054_2017_1728_MOESM3_ESM.docx]

Table 3: Results of subgroup analysis and sensitivity analyses for mortality and overall complication rates

|  | Overall complication rates | | Long-term mortality | | Short-term mortality | |
| --- | --- | --- | --- | --- | --- | --- |
|  | N(n) | RR(95% CI) | N(n) | RR(95% CI) | N(n) | RR (95%CI) |
| **Subgroup analyses** | | | | |  |  |
| Type of patients | | | | | | |
| High-risk patients | 10 (1722) | 0.65 [0.56, 0.76]* | 12 (3819) | 0.57 [0.36, 0.89]* | 12 (3698) | 0.73 [0.58, 0.91]* |
| Non high-risk patients | 21 (2240) | 0.84 [0.74, 0.96]* | 22 (1983) | 0.78 [0.46, 1.33] | 22 (1983) | 0.80 [0.49, 1.31] |
| ERP | | | | | | |
| In ERP | 9(1111) | 0.75 [0.58, 0.96]* | 9 (1040) | 0.87 [0.38, 1.99] | 9 (1040) | 0.71 [0.30, 1.69] |
| Not in ERP | 22 (2851) | 0.75 [0.67, 0.85]* | 25(4762) | 0.88[0.75, 1.04] | 25(4762) | 0.71 [0.53, 0.94]* |
| monitor | | | | | | |
| PAC | 5 (484) | 0.80 [0.57, 1.12] | 7 (2565) | 0.89 [0.73, 1.08] | 7 (2565) | 0.36 [0.14, 0.96]* |
| OEDM | 10(1064) | 0.75 [0.59, 0.95]* | 10 (993) | 0.96 [0.41, 2.25] | 10 (993) | 0.84 [0.31, 2.24] |
| Pulse contour analysis monitor^1^ | 10 (1622) | 0.75 [0.64, 0.87]* | 12 (1683) | 0.74 [0.52, 1.04] | 12 (1683) | 0.74 [0.53, 1.05] |
| Others^2^ | 6 (792) | 0.73 [0.54, 1.00] | 5 (606) | 0.62 [0.28, 1.41] | 5 (606) | 0.66 [0.31, 1.39] |
| Goal | | | | | | |
| CI^#^/DO_2_I | 7 (746) | 0.78 [0.63, 0.97]* | 9(2829) | 0.48 [0.25, 0.94]* | 9(2829) | 0.49 [0.25, 0.94]* |
| Optimal SV | 14 (2039) | 0.80 [0.69, 0.93]* | 14 (2007) | 0.74 [0.51, 1.07] | 14 (2007) | 0.72 [0.49, 1.05] |
| SVV and other dynamic measures^3^ | 6 (560) | 0.64 [0.52, 0.79]* | 9 (662) | 0.78 [0.35, 1.76] | 9 (662) | 0.78 [0.35, 1.76] |
| Others^4^ | 4 (617) | 0.69 [0.43, 1.10] | 2 (349) | 0.49 [0.12, 1.91] | 2 (349) | 0.53 [0.12, 2.24] |
| Interventions | | | | | | |
| Fluids only | 12 (1349) | 0.75 [0.62, 1.00] | 14(1286) | 1.11 [0.52, 2.36] | 14(1286) | 1.05 [0.47, 2.33] |
| Fluids and inotropes | 19 (2613) | 0.76 [0.66, 0.86]* | 20 (4561) | 0.63 [0.44, 0.89]* | 20 (4561) | 0.65 [0.47, 0.89]* |
| **Sensitivity analyses** | | | | |  |  |
| low risk of bias | 20(2892) | 0.78 [0.70, 0.87]* | 16 (4280) | 0.90 [0.76, 1.06] | 16 (4280) | 0.80 [0.64, 1.00] |
| sample size 100 or greater | 19 (3210) | 0.79 [0.69, 0.89]* | 17 (4589) | 0.89 [0.76, 1.06] | 15 (4387) | 0.82 [0.65, 1.02] |
| colorectal surgery | 9 (1059) | 0.89 [0.75, 1.06] | 6 (654) | 0.90 [0.35, 2.29] | 6 (654) | 0.72 [0.23, 2.20] |

RR=risk ratio; CI=95% confidence interval; ERP=enhanced recovery protocol; N=number of studies; n=number of participants; PAC= pulmonary artery catheter; OEDM=oesophageal Doppler monitor; CI^#^=cardiac index; DO_2_I=oxygen delivery index; SV=stroke volume; SVV=stroke volume variation

1 self-calibrating/ calibrated pulse contour analysis monitor for example, Vigileo/Flotrac, LiDCO, PiCCO.

2 arterial line monitoring equipment, central line and arterial line sampling, pulse oximeter, and other non-invasive monitors.

3 pulse pressure variation (PPV), variation in arterial pulse pressure and pleth variability index (PVI)

4 mixed venous oxygen saturation, oxygen extraction ratio or lactate

*statistically significant.
