# Supplementary material for: Effect of perioperative goal-directed hemodynamic therapy on postoperative recovery following major abdominal surgery—a systematic review and meta-analysis of randomized controlled trials
Source: Crit Care. 2017 Jun 12;21:141. doi: 10.1186/s13054-017-1728-8 (PMC5467058; doi:10.1186/s13054-017-1728-8)
Supplement: Supplementary file 4 — Meta-regression analysis for long-term mortality based on type of patients (high-risk versus non-high-risk), type of monitoring used, type of interventions (fluids versus fluids and inotropes), therapeutic goals, and whether in context with enhanced recovery programs (ERPs). RR Risk ratio. (PDF 100 kb) [file 13054_2017_1728_MOESM4_ESM.pdf]

Type of patients

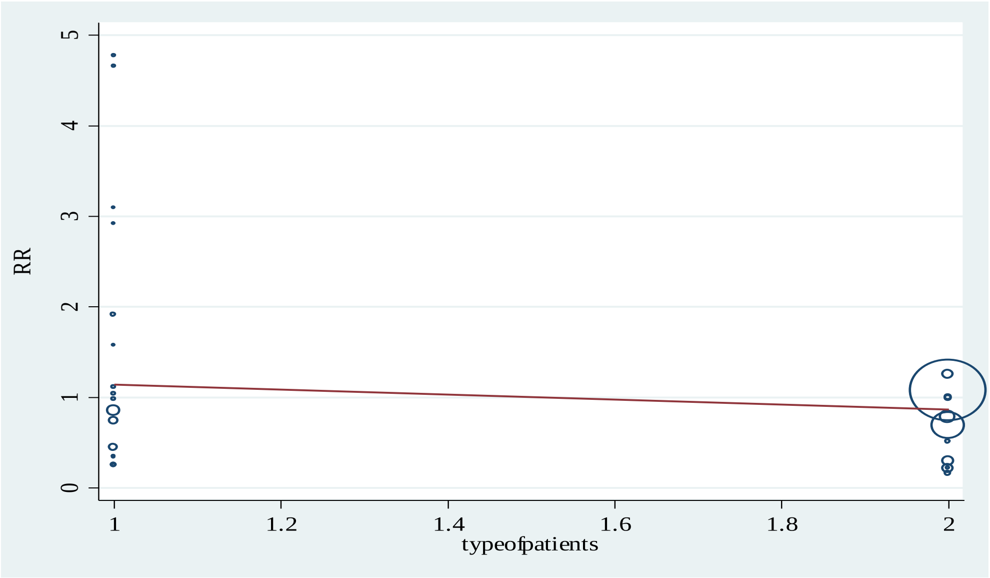

Type of monitor

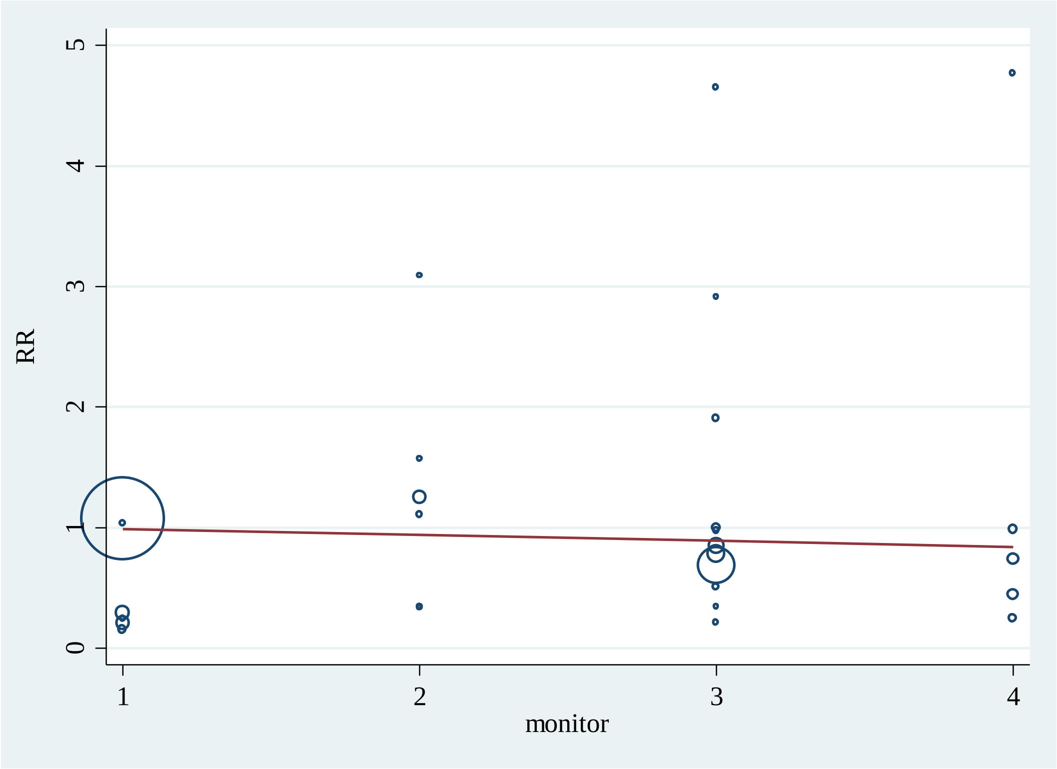

Therapeutic goal

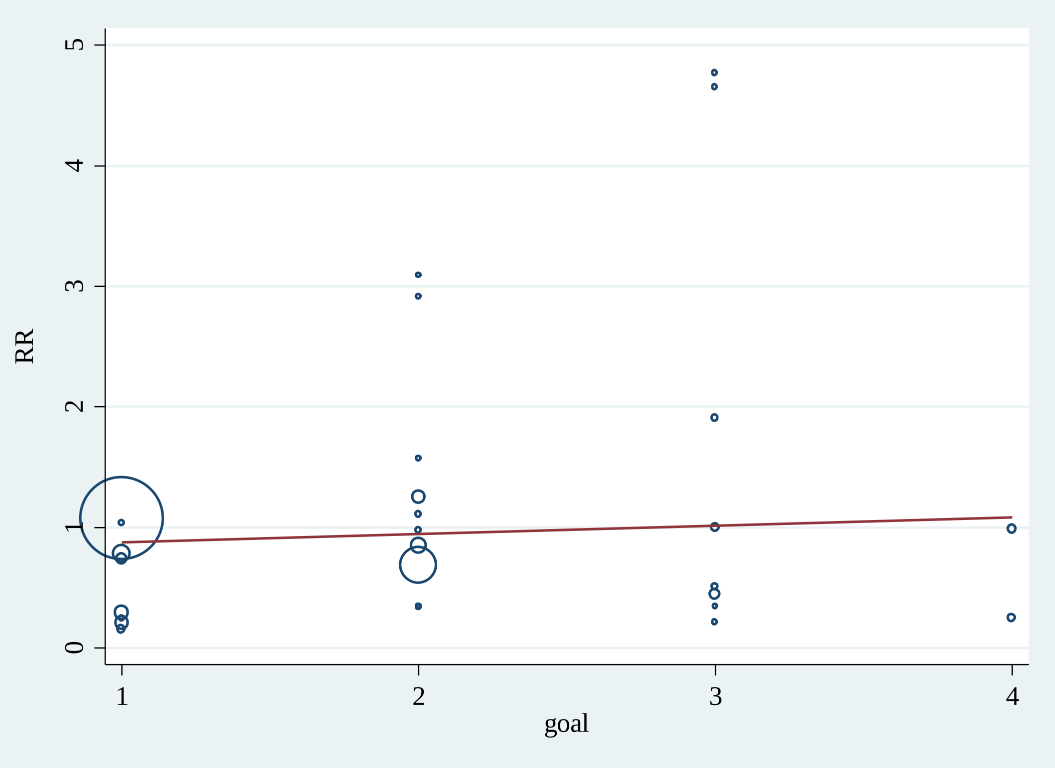

Interventions

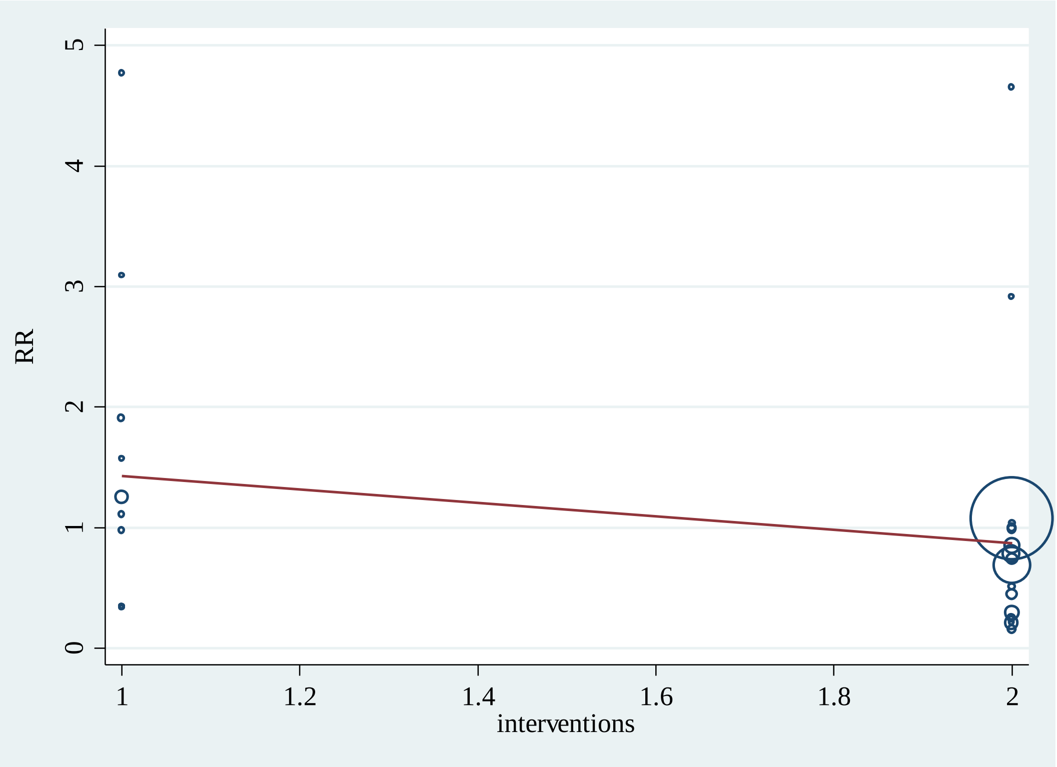

ERP

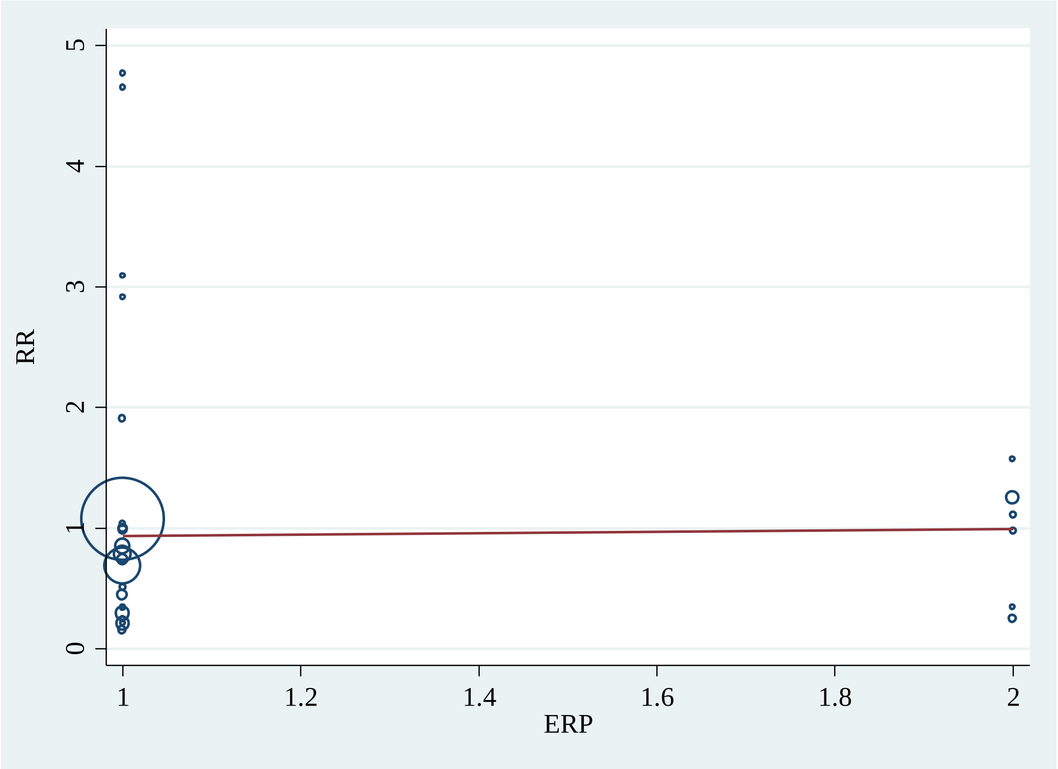

Additional file 4: Meta-regression analysis for long-term mortality based on type of patients (high-risk versus non high-risk), type of monitoring used, type of interventions (fluids versus fluids and inotropes), therapeutic goals, and whether or not in context with enhanced recovery programmes (ERP). RR: risk ratio.
