# Supplementary figures and images for: Effect of perioperative goal-directed hemodynamic therapy on postoperative recovery following major abdominal surgery—a systematic review and meta-analysis of randomized controlled trials
Source: Crit Care. 2017 Jun 12;21:141. doi: 10.1186/s13054-017-1728-8 (PMC5467058; doi:10.1186/s13054-017-1728-8)

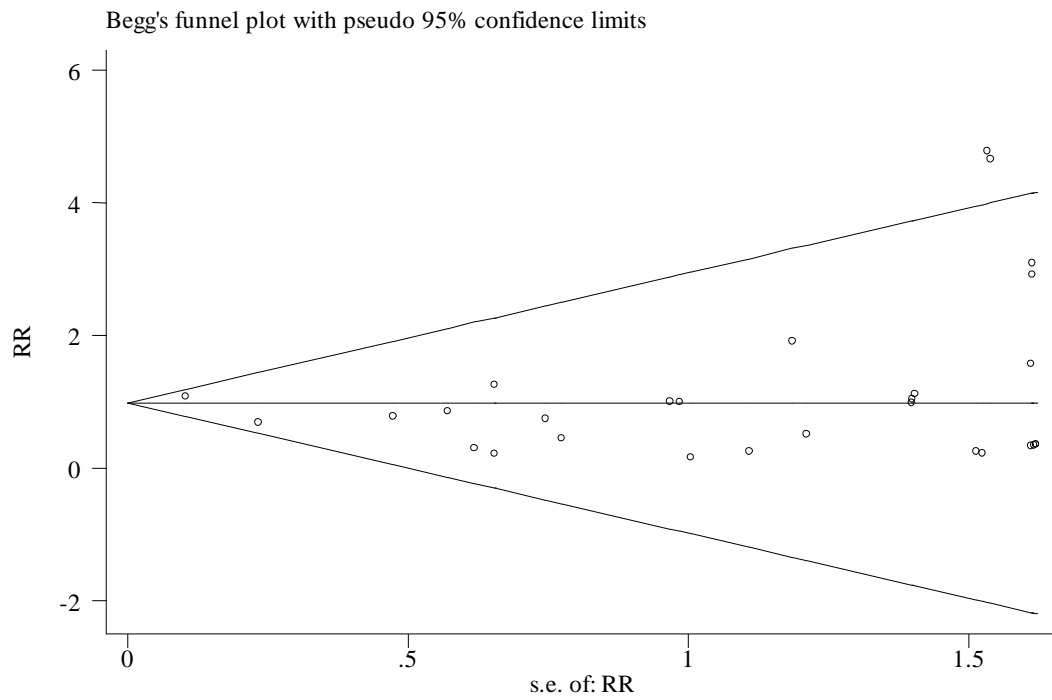

Additional file 5: Begg's publication funnel plots onlong-term mortality. RR=risk ratio

Supplement: Supplementary file 5 — Begg’s publication funnel plots on long-term mortality. RR Risk ratio. (PDF 127 kb) [file 13054_2017_1728_MOESM5_ESM.pdf]

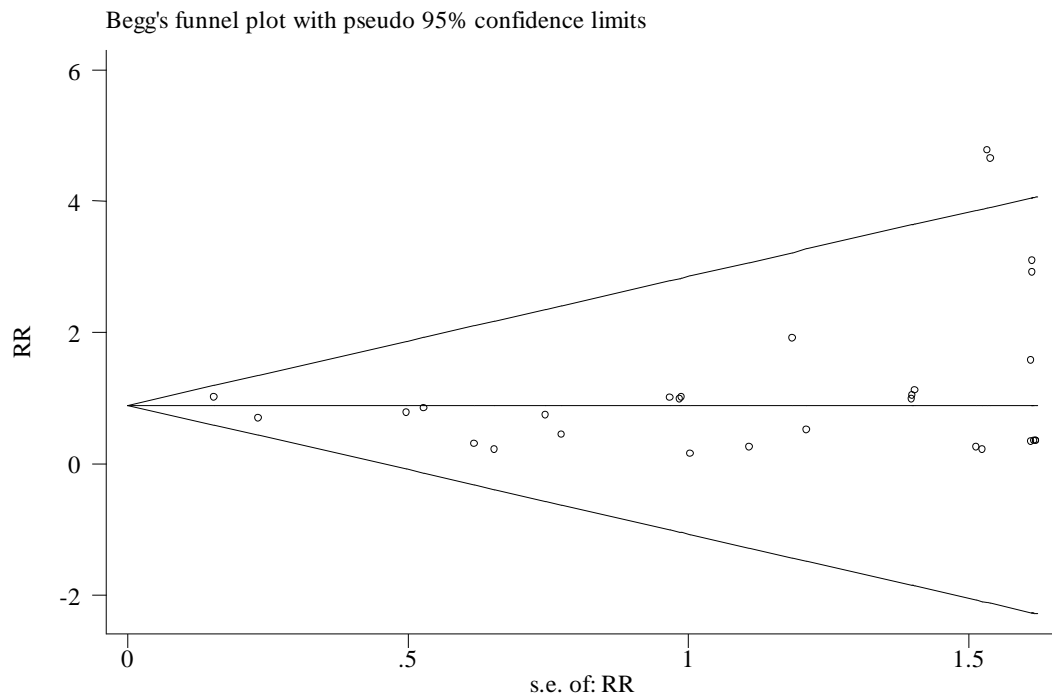

Additional file 7: Publication funnel plots for short-term mortality. RR: risk ratio

Supplement: Supplementary file 7 — Publication funnel plots for short-term mortality. RR Risk ratio. (PDF 82 kb) [file 13054_2017_1728_MOESM7_ESM.pdf]

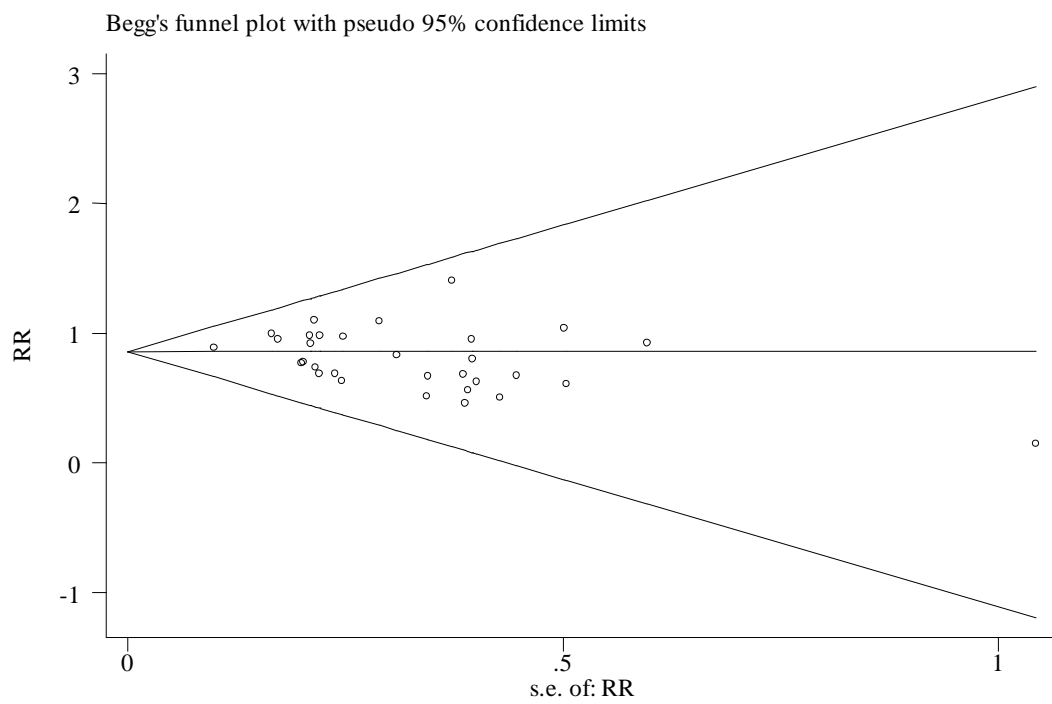

Additional file 9 : Begg's publication funnel plots on overall complication rates.

RR=risk ratio

Supplement: Supplementary file 9 — Begg’s publication funnel plots on overall complication rates. RR Risk ratio. (PDF 128 kb) [file 13054_2017_1728_MOESM9_ESM.pdf]
