# Supplementary material for: Effect of perioperative goal-directed hemodynamic therapy on postoperative recovery following major abdominal surgery—a systematic review and meta-analysis of randomized controlled trials
Source: Crit Care. 2017 Jun 12;21:141. doi: 10.1186/s13054-017-1728-8 (PMC5467058; doi:10.1186/s13054-017-1728-8)
Supplement: Supplementary file 6 — Meta-regression analysis for short-term mortality. ERP Enhanced recovery program. (PDF 99 kb) [file 13054_2017_1728_MOESM6_ESM.pdf]

Type of patients

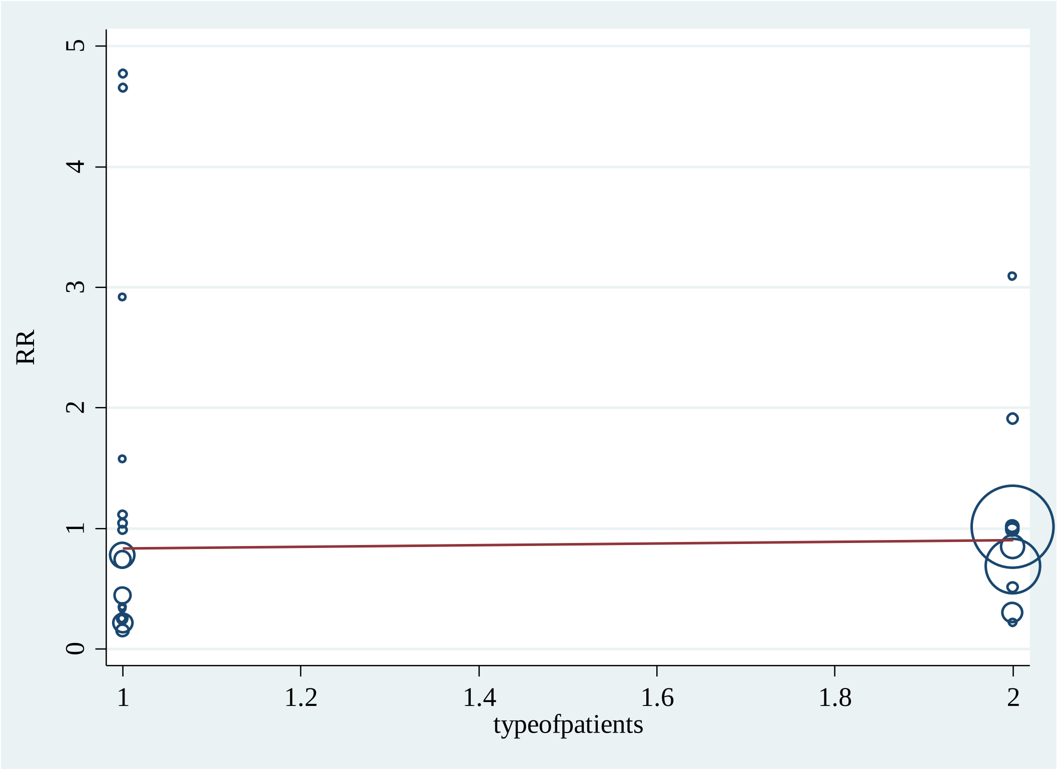

Type of monitor

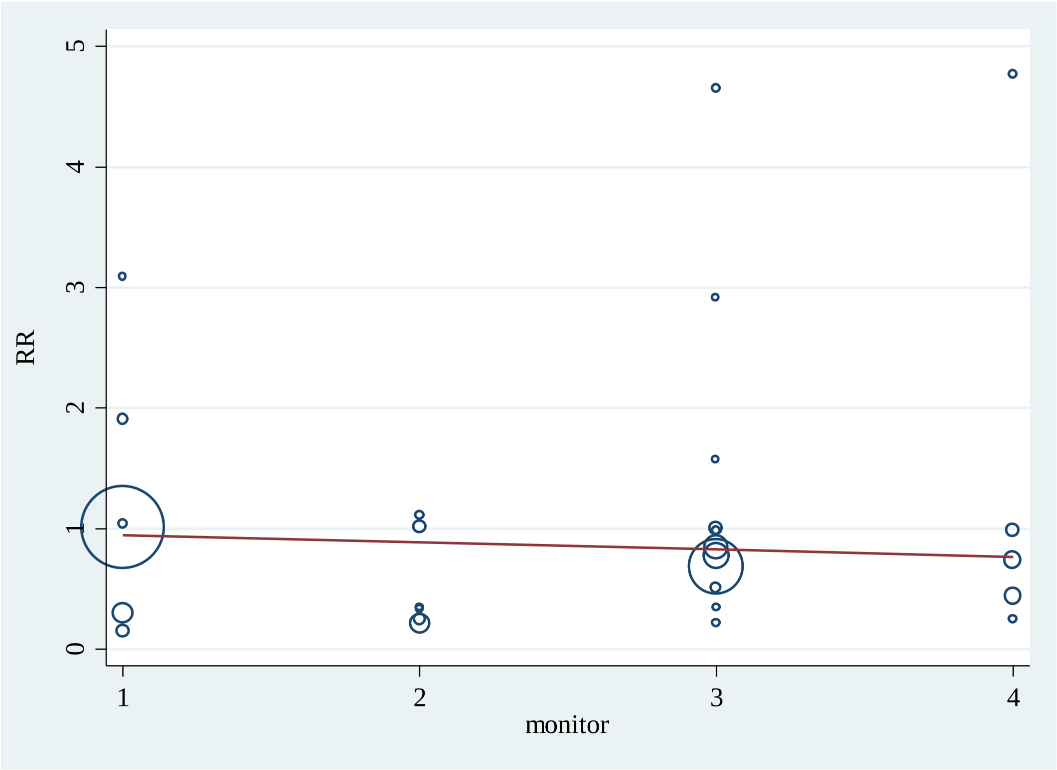

Therapeutic goal

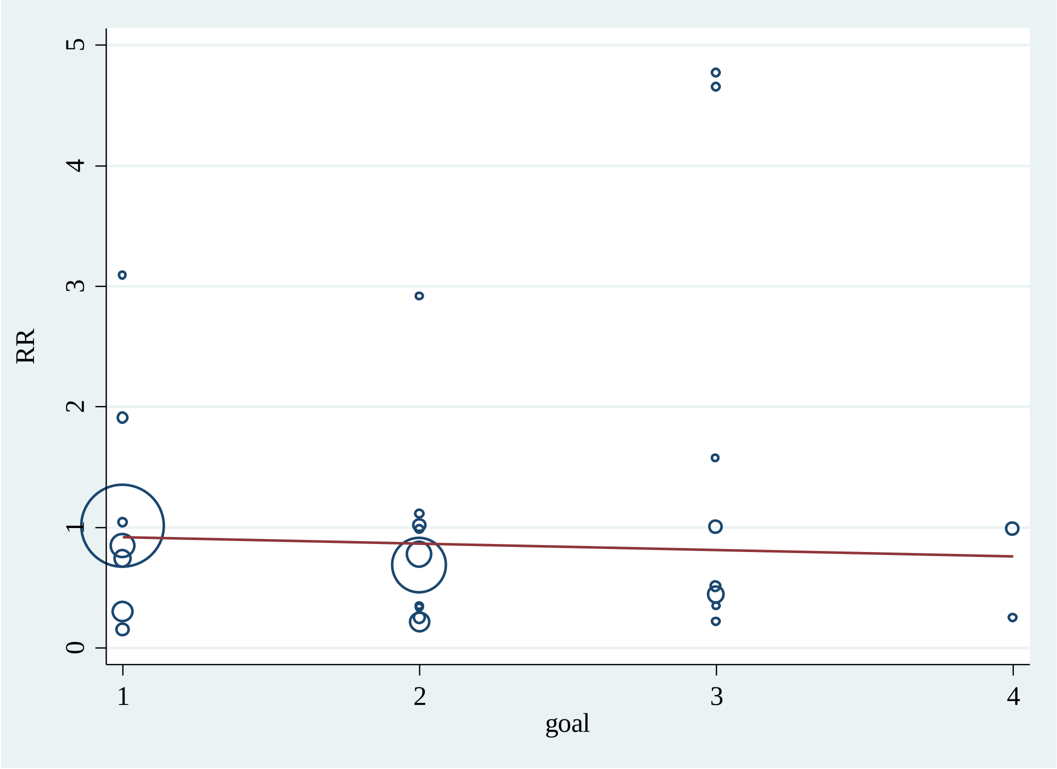

Type of interventions

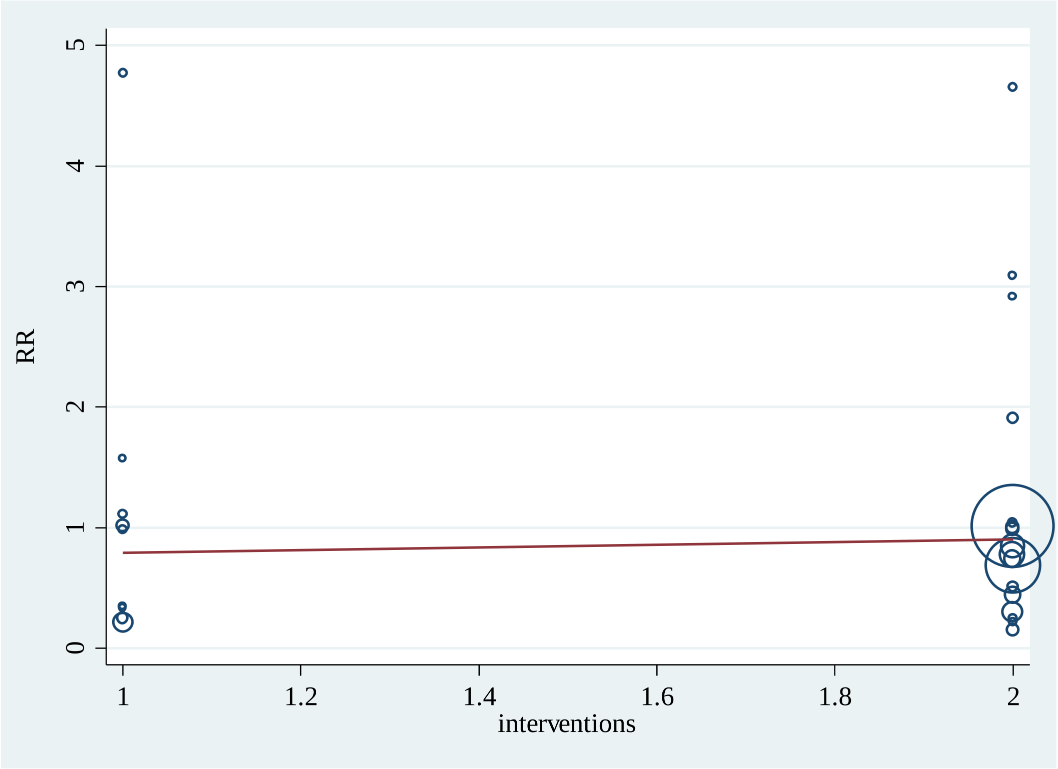

ERP

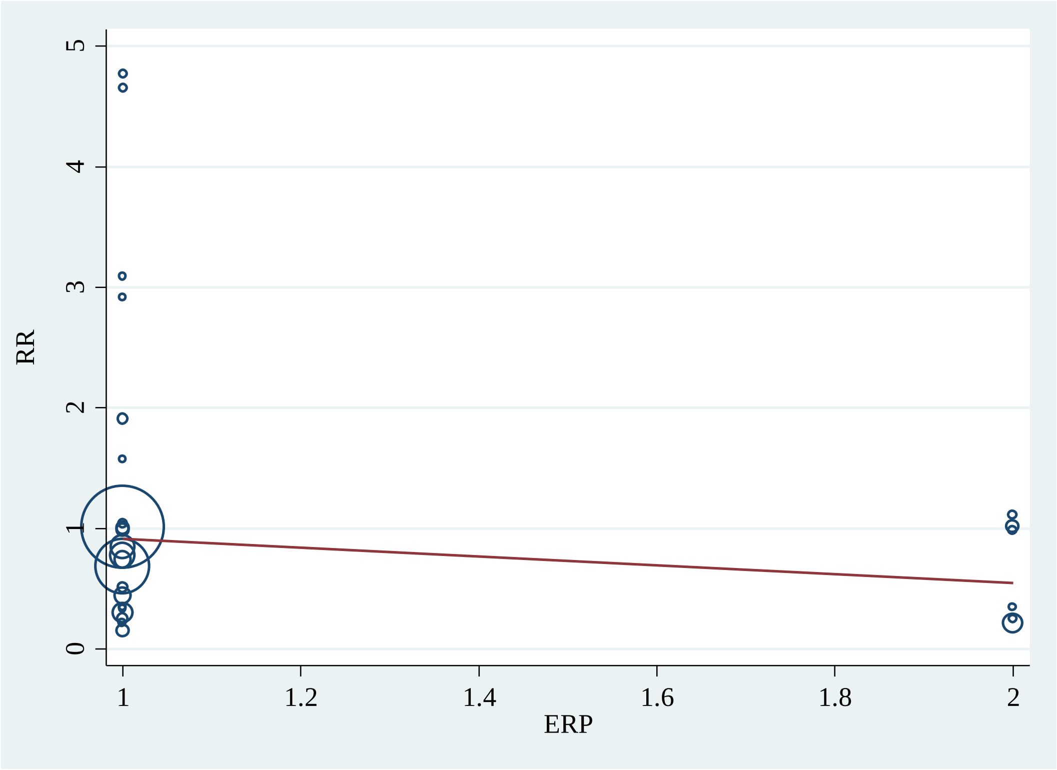

Additional file 6 : Meta-regression analysis for short-term mortality. ERP: enhanced recovery programmes.
