# Supplementary material for: Effect of perioperative goal-directed hemodynamic therapy on postoperative recovery following major abdominal surgery—a systematic review and meta-analysis of randomized controlled trials
Source: Crit Care. 2017 Jun 12;21:141. doi: 10.1186/s13054-017-1728-8 (PMC5467058; doi:10.1186/s13054-017-1728-8)
Supplement: Supplementary file 8 — Meta-regression analysis for overall complication rates. RR Risk ratio, ERP Enhanced recovery program. (PDF 18 kb) [file 13054_2017_1728_MOESM8_ESM.pdf]

Type of patients

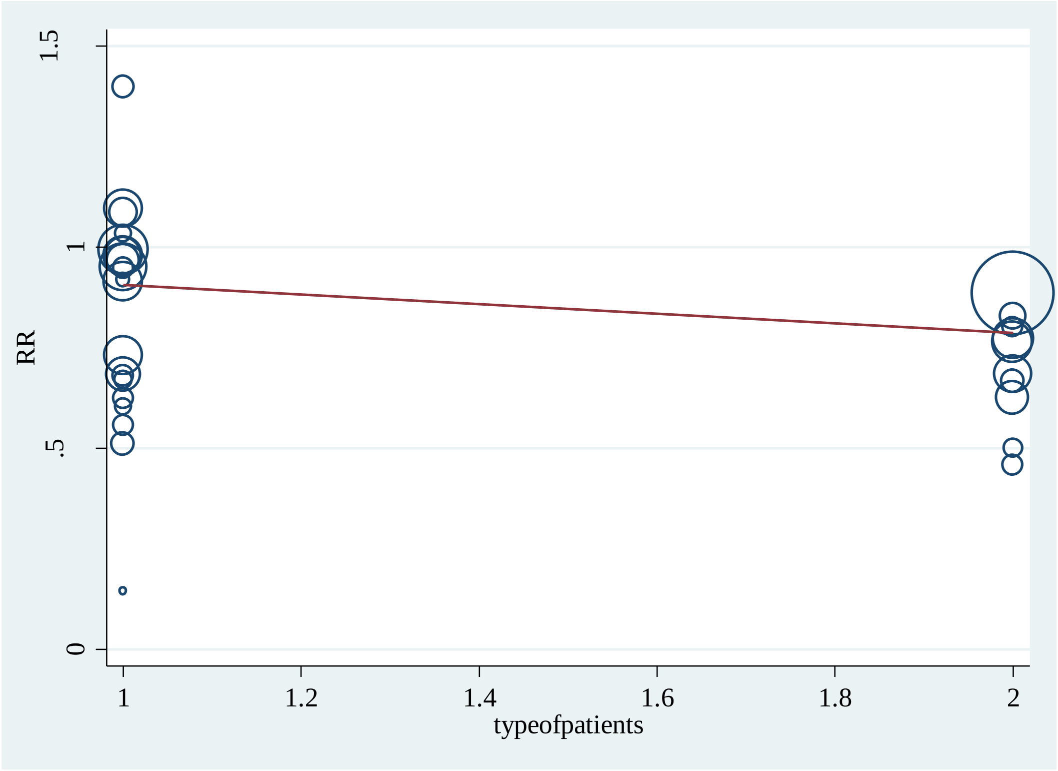

Monitor

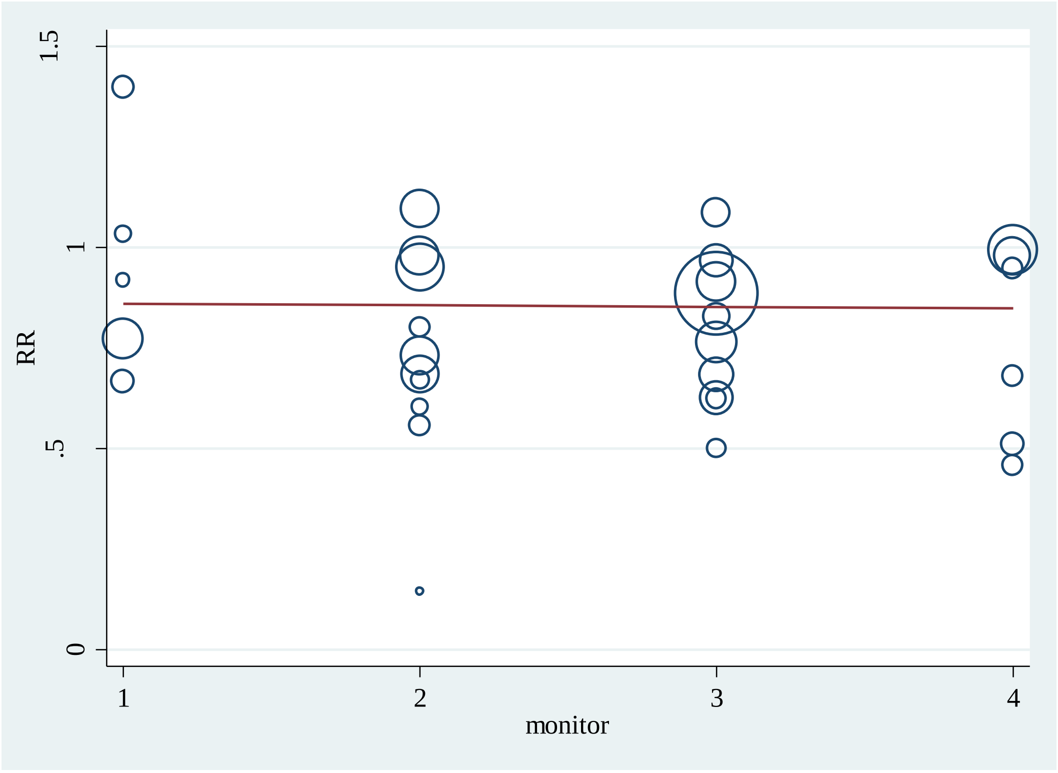

Therapeutic Goals

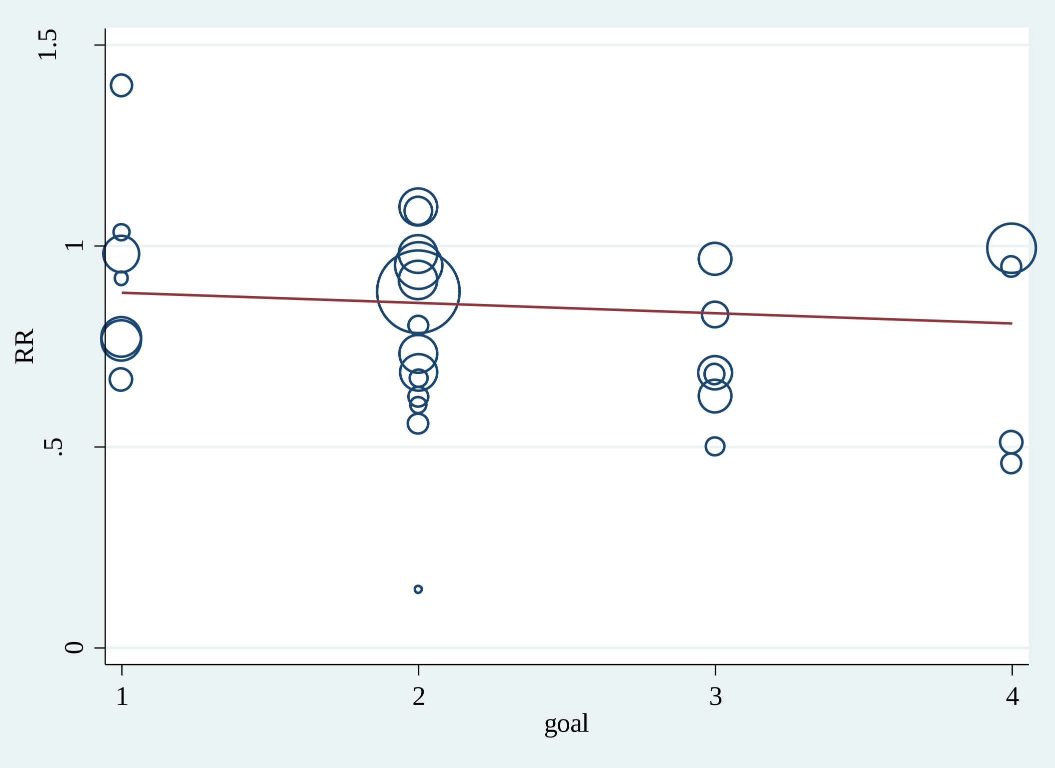

Interventions

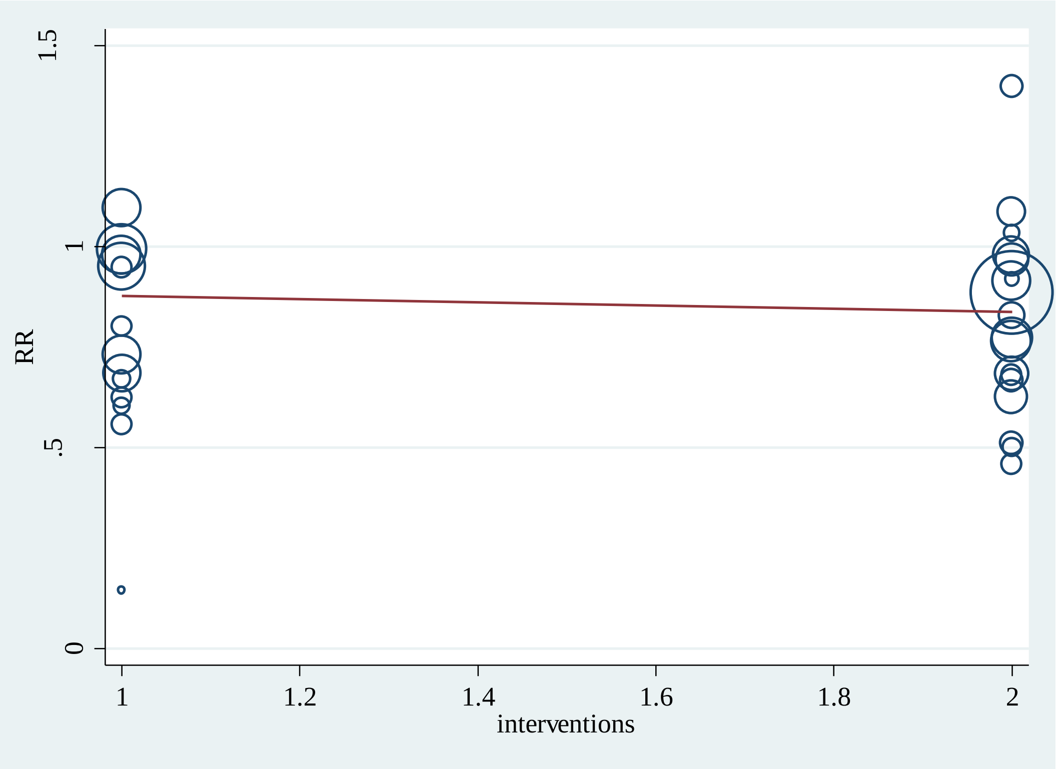

ERP

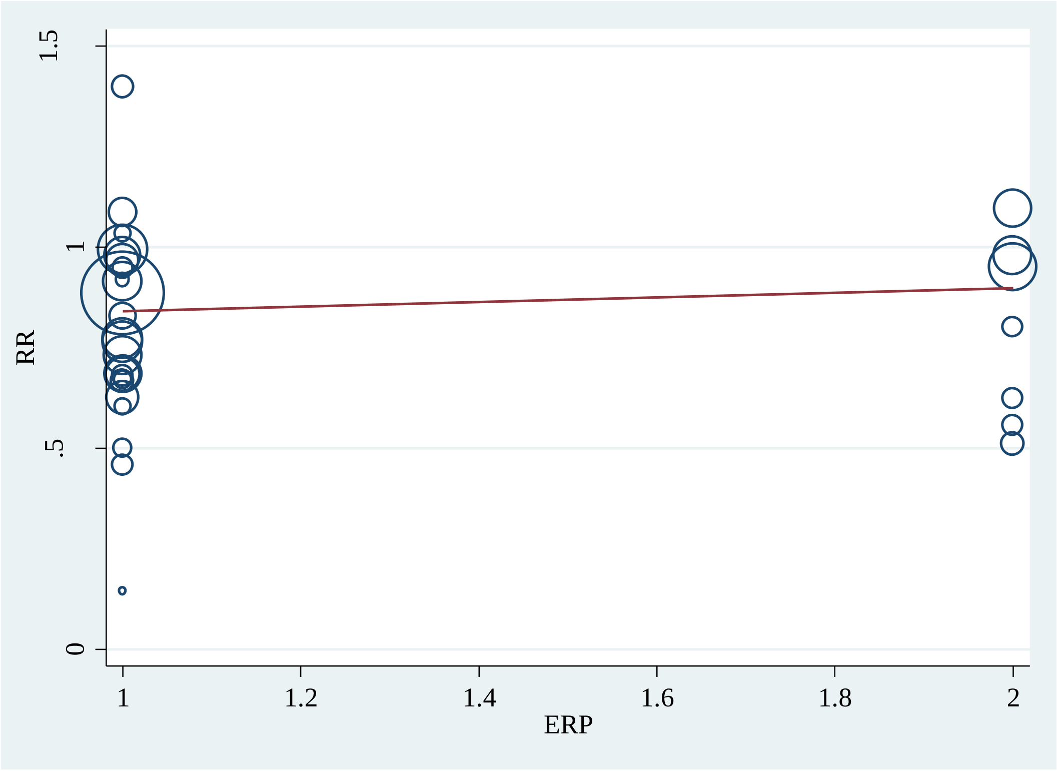

Additional file 8: Meta-regression analysis for overall complication rates.

RR=risk ratio; ERP=enhanced recovery programmes.
