# Supplementary material for: Effect of perioperative goal-directed hemodynamic therapy on postoperative recovery following major abdominal surgery—a systematic review and meta-analysis of randomized controlled trials
Source: Crit Care. 2017 Jun 12;21:141. doi: 10.1186/s13054-017-1728-8 (PMC5467058; doi:10.1186/s13054-017-1728-8)
Supplement: Supplementary file 10 — Begg’s publication funnel plots on time to first flatus pass (a), time to bowel movement (b), and time to tolerate oral diet (c). WMD Weighted mean difference. (PDF 54 kb) [file 13054_2017_1728_MOESM10_ESM.pdf]

A: Time to first flatus pass

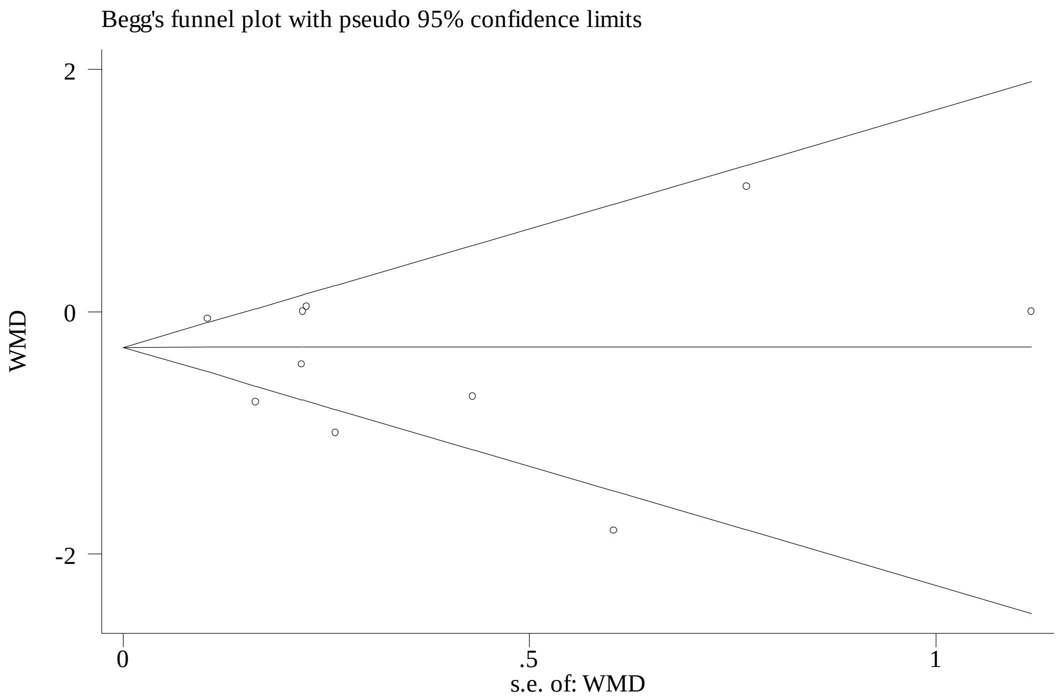

B: Time to first bowel movement

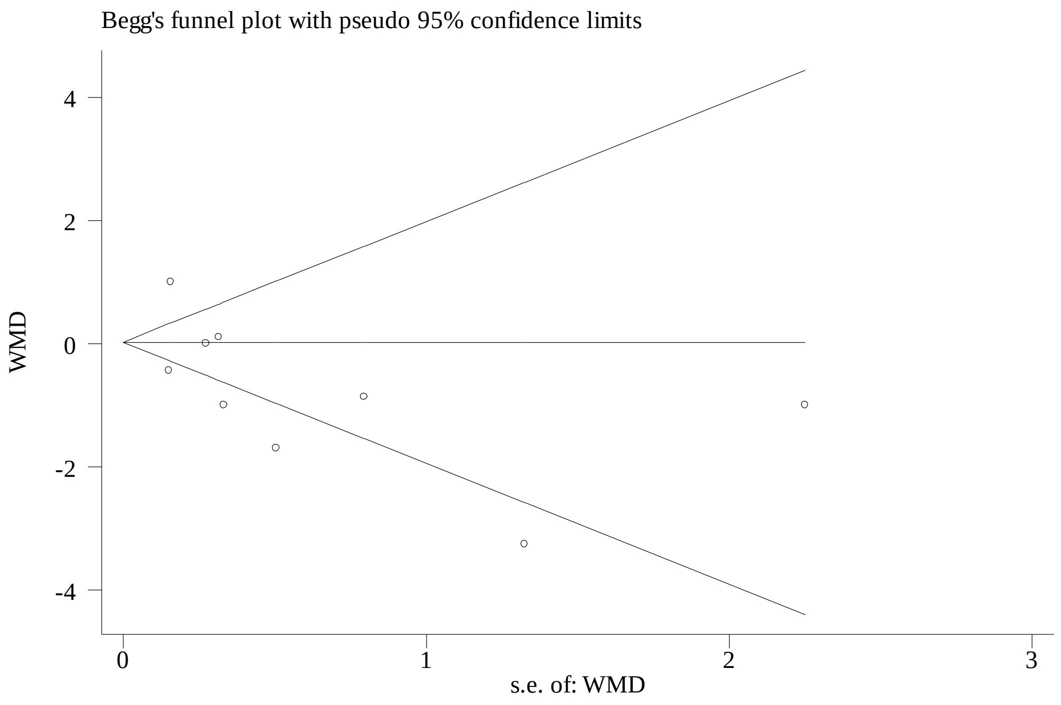

C:Time to tolerate oral diet

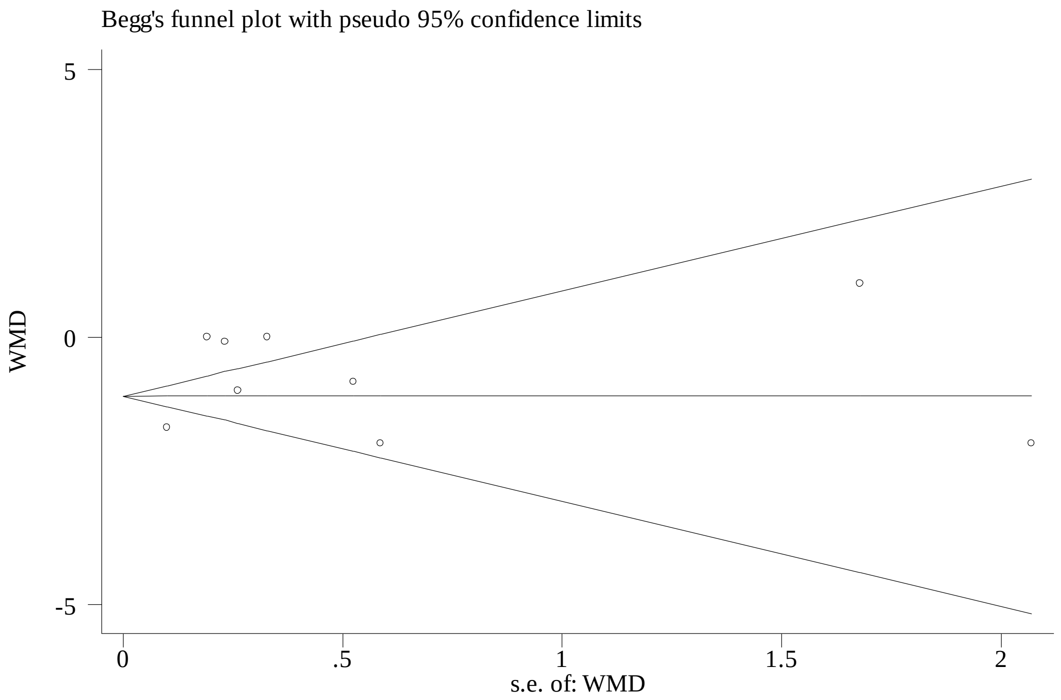

Additional file 10: Begg’s publication funnel plots on time to first flatus pass (A), time to bowel movement (B), and time to tolerate oral diet (C). WMD: weighted mean differences.
